# Dynamic Active Site Evolution in Lanthanum‐Based Catalysts Dictates Ethane Chlorination Pathways

**DOI:** 10.1002/anie.202505846

**Published:** 2025-06-26

**Authors:** Yuting Li, Haifeng Qi, Zihan Zhu, Xia Wu, Nicholas F. Dummer, Stuart H. Taylor, Lei Ma, Xiaofeng Yang, Qinggang Liu, Graham J. Hutchings, Yanqiang Huang

**Affiliations:** ^1^ State Key Laboratory of Catalysis Dalian Institute of Chemical Physics Chinese Academy of Sciences Dalian 116023 China; ^2^ University of Chinese Academy of Sciences Beijing 100049 China; ^3^ Max Planck‐Cardiff Centre on the Fundamentals of Heterogeneous Catalysis FUNCAT Cardiff Catalysis Institute Translational Research Hub Cardiff University Maindy Road Cardiff CF24 4HQ UK; ^4^ Chemical Engineering and Resource Utilization Northeast Forestry University Harbin 150040 China

**Keywords:** Ethane chlorination, Lanthanum oxychloride, Natural gas, Over chlorination, Polyvinyl chloride, Structural evolution

## Abstract

Radical‐mediated chlorination of ethane presents a low‐carbon alternative for polyvinyl chloride (PVC) synthesis, yet selectivity toward 1,2‐dichloroethane remains challenged by uncontrolled over‐chlorination. Lanthanum oxychloride (LaOCl) has emerged as a promising catalyst, but its structural dynamics under Cl_2_‐rich conditions and the origin of selectivity loss remain elusive. Here, we integrate advanced spectroscopic techniques with theoretical calculations to address this knowledge gap. Our findings unveil a sequential LaOCl → LaCl_3_ transformation that dictates product distribution shifting from 1,2‐dichloroethane to trichloroethane. Mechanistic insights reveal that surface hydroxyl groups, generated during catalyst chlorination, promote bidentate adsorption of 1,2‐dichloroethane via hydrogen‐bond networks, thereby activating C─Cl over‐chlorination. Additionally, by employing Al_2_O_3_‐supported LaCl_3_ model catalysts, the size‐dependent chlorophilicity of the LaCl_3_ species is demonstrated. The bonding of interfacial oxygen with monolayer‐dispersed LaCl_3_ species generates empty 4f‐states above the Fermi level, creating strong Lewis acid sites that stabilize Cl radicals and selectively convert chloroethane to 1,2‐dichloroethane. In contrast, aggregated nanoparticles are inactive due to their inability to stabilize chlorine radical. Our findings establish important structure sensitivity in lanthanum‐catalyzed chlorination and provide guiding principles for catalyst design, highlighting the importance of stabilizing metastable LaOCl*_x_* species and modulating surface hydroxyl chemistry to overcome selectivity limitations.

## Graphical abstract

LaOCl‐catalyzed C_2_H_6_ chlorination offers a low‐carbon route for polyvinyl chloride synthesis, yet structural evolution occurs under Cl_2_ conditions. We establish structural sensitivity in La‐catalyzed chlorination, and elucidate the size‐dependent chlorophilicity of LaCl_3_ and the role of hydroxyl groups in overchlorination, highlighting stabilization of LaOCl and hydroxyl chemistry modulation as key design principles to break selectivity limitations.

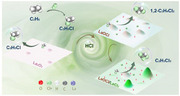

## Introduction

Polyvinyl chloride (PVC) is the third most manufactured polymer globally, with annual production exceeding 50 million tons.^[^ ^1^, ^2^ ^]^ Such widespread application has driven significant industrial interest in developing more efficient PVC production methods.^[^ ^3^, ^4^, ^5^, ^6^ ^]^ PVC can be readily synthesized from vinyl chloride monomer (VCM) through a radical‐based polymerization process.^[^ ^7^, ^8^ ^]^ However, the current coal‐to‐acetylene and petroleum‐to‐ethylene routes for VCM synthesis face critical sustainability limitations.^[^ ^9^, ^10^, ^11^, ^12^, ^13^, ^14^ ^]^ The coal‐to‐acetylene route requires energy‐demanding calcium carbide production at >2000 °C,^[^ ^15^ ^]^ resulting in 8–12 tons of CO_2_ emissions per ton of VCM.^[^ ^16^ ^]^ Similarly, petroleum‐derived routes depend on high‐temperature steam‐cracking operations (>500 °C) for ethylene (C_2_H_4_) generation,^[^ ^17^ ^]^ emitting 3–4 tons of CO_2_ emissions per VCM ton.^[^ ^18^, ^19^ ^]^ These carbon‐intensive methodologies collectively represent a notable portion of global chemical industry emissions, highlighting the urgent need for developing alternative feedstocks for sustainable VCM synthesis.

Natural gas‐derived ethane (C_2_H_6_) is a promising candidate for VCM production due to its abundance and low carbon footprint.^[^ ^20^, ^21^, ^22^, ^23^ ^]^ Over the past few decades, the C_2_H_6_ oxychlorination process, which involves co‐feeding HCl, O_2_, and Cl_2_ in a single reactor, has been extensively researched.^[^ ^24^, ^25^, ^26^ ^]^ However, the C_2_H_6_ oxychlorination process requires high temperatures (450–550 °C) to activate the relatively unreactive C_2_H_6_.^[^ ^27^, ^28^ ^]^ The limited selectivity for VCM, due to C_2_H_4_ formation, along with considerable carbon loss in the form of carbon oxides due to the use of O_2_, poses significant challenges.^[^ ^29^ ^]^ Compared with the oxychlorination process, reacting C_2_H_6_ with Cl_2_ is an excellent alternative strategy for VCM production.^[^ ^30^ ^]^ Previous work by Olsbye et al. indicated that thermal‐induced dissociation of Cl_2_ produces chlorine radicals (Cl·), which activates C_2_H_6_ at mild temperatures (<300 °C).^[^ ^31^ ^]^ Notably, the radical‐driven chlorination of C_2_H_6_ yields almost exclusively ethyl chloride (C_2_H_5_Cl) even at high conversion of up to 50%.^[^ ^32^ ^]^ A catalyst can be designed to target the chlorination of C_2_H_5_Cl into 1,2‐dichloroethane (1,2‐C_2_H_4_Cl_2_), which can then be thermally cracked into VCM. However, despite showing promise, chlorination of C_2_H_6_ directly to 1,2‐C_2_H_4_Cl_2_ has yet to be implemented due to inherent challenges in achieving acceptable product selectivity. Gas‐phase Cl radical‐mediated hydrogen abstraction favors the formation of the 1‐chloroethyl radical (15 kJ mol^−1^ lower in energy than the 2‐chloroethyl isomer), leading to the undesired production of 1,1‐C_2_H_4_Cl_2_.^[^ ^31^ ^]^ The low activation barriers for sequential H‐abstraction and Cl· transfer further exacerbate competing pathways, including over‐chlorination and thermal dehydrochlorination.

Recent studies by Zichittella and Pérez‐Ramírez have demonstrated that these challenges can be addressed by activating the C_2_H_5_Cl intermediate on rare‐earth oxychloride catalysts.^[^ ^30^ ^]^ Among these catalysts, LaOCl exhibits exceptional promise due to its unique chlorophilic properties, which facilitate efficient Cl_2_ dissociation. This enables the selective conversion of C_2_H_5_Cl to 1,2‐C_2_H_4_Cl_2_, achieving 80% selectivity at 20% C_2_H_6_ conversion under a C_2_H_6_/Cl_2_ ratio of 6:3 (v/v). Nevertheless, a critical 1,2‐C_2_H_4_Cl_2_ selectivity limitation emerges at elevated C_2_H_6_ conversion. Notably, LaOCl is prone to progressive surface chlorination, leading to the formation of LaCl_3_, under Cl_2_‐rich conditions.^[^ ^33^, ^34^, ^35^ ^]^ While the structural sensitivity of C_2_H_6_ chlorination to La sites can be mitigated by lowering Cl_2_ feed concentrations, the impact of these dynamic active sites under industrially relevant conditions remains unresolved. Addressing the mechanistic gap between the electronic states of La sites and their chlorination behavior is imperative to reduce selectivity constraints inherent with La‐based catalysts.

In this work, we investigate the structural evolution of La_2_O_3_ catalysts under Cl_2_‐rich conditions (C_2_H_6_:Cl_2_ = 4:9, v/v), aiming to shed light on the complex C_2_H_6_ chlorination reaction network on oxychloride‐based materials. Our in‐depth characterization reveals a sequential La_2_O_3_ → LaOCl → LaCl_3_ transformation, driven by progressive chlorination. This structural evolution directly governs product distribution, with chlorination selectivity shifting from C_2_H_5_Cl to 1,2‐C_2_H_4_Cl_2_, and ultimately to trichloroethane (C_2_H_3_Cl_3_). By combining Al_2_O_3_‐supported LaCl_3_ model catalysts and theoretical calculations, we elucidate the size‐dependent chlorophilicity of LaCl_3_ and the role of hydroxyl groups in promoting over‐chlorination. These findings establish chlorination‐driven phase dynamics as the central determinant of product selectivity, offering a blueprint for designing selective catalysts via precise control of metal speciation and surface microenvironment.

## Results and Discussion

### Structural Evolution of the La_2_O_3_ Under Cl_2_‐Rich Conditions

**Figure 1:**
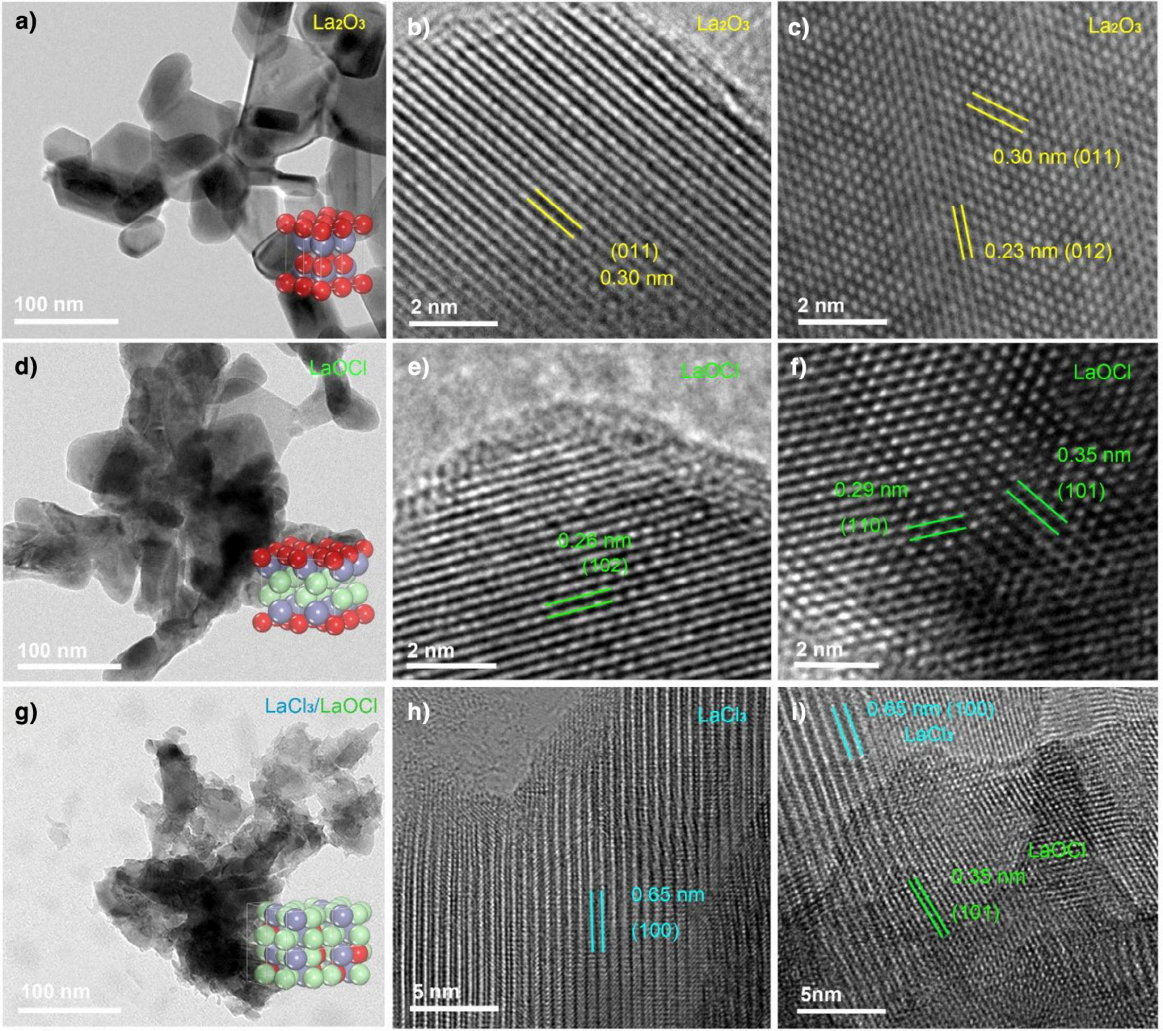
Time‐resolved HAADF‐STEM analysis of La_2_O_3_ structural evolution under Cl_2_‐rich conditions. a)–c) Pristine La_2_O_3_ catalyst. d)–f) Intermediate stage after 55 min reaction. g)–i) Final stage after 770 min reaction.

The La_2_O_3_ catalyst was synthesized via an ammonia precipitation method followed by high‐temperature calcination at 800 °C. To investigate structural evolution under industrially relevant conditions (C_2_H_6_:Cl_2_ = 4:9, v/v), we conducted time‐resolved characterization combining high‐resolution transmission electron microscopy (HRTEM) and energy‐dispersive spectroscopy (EDS). These complementary techniques provide critical insights into morphological transformations and chlorination dynamics during the catalytic process (Figure S1). Initial HRTEM characterization (Figure 1a–c) reveals that the pristine catalyst exhibited well‐defined lattice spacings of 0.30 and 0.23 nm, corresponding to the (011) and (012) planes of hexagonal La_2_O_3_, respectively.^[^ ^36^, ^37^ ^]^ Upon exposure to the Cl_2_‐rich conditions, time‐dependent structural degradation was observed (Figure 1d). When the reaction proceeded to 55 min, the emergence of 0.26 nm lattice fringes (Figure 1e) are observable, matching the (102) planes of tetragonal LaOCl.^[^ ^38^ ^]^ The intermediate chlorination state of LaOCl is further confirmed by Figure 1f which displays characteristic spacings of 0.29 and 0.35 nm, corresponding to Miller indices (hkl) of 110 and 101, respectively.^[^ ^39^ ^]^ The structural evolution progresses more extensively at extended reaction times (Figure 1g). After 770 min, HRTEM images (Figure 1h,i) reveal 0.65 nm lattice spacing corresponding to the (100) planes of hexagonal LaCl_3_, demonstrating further chlorination of the LaOCl phase. However, residual 0.35 nm spacings corresponding to LaOCl (101) planes persist in localized regions (Figure 1i), revealing incomplete conversion of the intermediate phase. This behavior may be attributed to the higher activation energy required for complete oxygen substitution in the LaOCl lattice compared to initial La_2_O_3_ chlorination. Complementary EDS analysis (Figure S2) quantitatively tracked the chlorination progression, showing a monotonic increase in Cl/La atomic ratios from 0 (initial) to 1 ± 0.04 (770 min).

**Figure 2:**
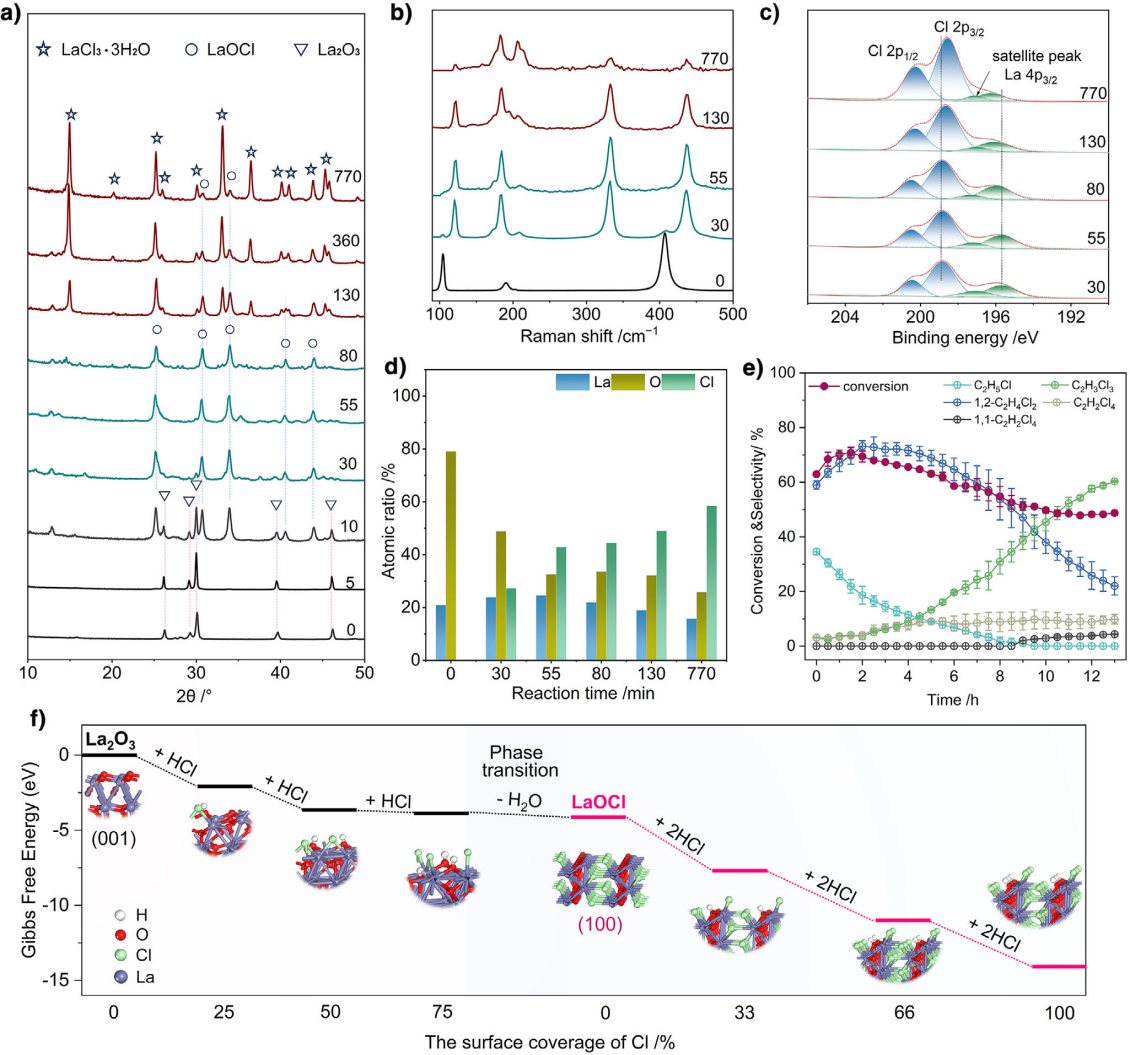
Structural changes of the La_2_O_3_ catalyst under reaction gas atmosphere at 260 °C. Time‐resolved XRD patterns a) and Raman spectra b). The numbers in the figures represent the reaction time of the catalyst in minutes. c) Time‐resolved XPS Cl 2p and La 4p_2/3_ spectra. d) Changes in surface atomic contents. e) Conversion and selectivity of C_2_H_6_ chlorination over the La_2_O_3_ catalyst. Reaction conditions: C_2_H_6_/Cl_2_/N_2_ = 4:9:87, 260 °C, WHSV = 2000 mL h^−1^ g^−1^. f) Gibbs free energy change during the surface chlorination of La_2_O_3_.

The phase evolution of La_2_O_3_ during chlorination was further investigated through analysis of synergistic X‐ray diffraction (XRD) and Raman spectroscopy. Figure 2a presents time‐resolved XRD patterns tracking crystalline phase evolution. Initially, distinct diffraction peaks corresponding to hexagonal La_2_O_3_ dominate the profile.^[^ ^34^ ^]^ At 55 min, reflections assignable to tetragonal LaOCl emerge,^[^ ^30^ ^]^ indicating partial oxychlorination of La_2_O_3_, in agreement with HRTEM observations of LaOCl lattice formation. After 360 min, the XRD pattern transitions to a LaCl_3_·3H_2_O dominated profile, demonstrating progressive chlorine substitution at oxygen sites. Notably, the disappearance of LaOCl signatures at this stage suggests thermodynamic instability of the intermediate phase under sustained Cl_2_ exposure, driving structural degradation and conversion to LaCl_3_.

Raman spectroscopic analysis (Figure 2b) provides insight into atomic‐scale structural dynamics. The pristine La_2_O_3_ spectrum exhibits characteristic vibrational modes at 104.5 cm^−1^ (A_1g_ symmetry, La─O symmetric stretching), 189.7 cm^−1^ (*E*_g_ symmetry, O─La─O bending), and 405.8 cm^−1^ (multiphonon coupling), indicative of its hexagonal lattice.^[^ ^40^ ^]^ After 55 min of reaction, new bands emerge at 184 cm^−1^ (Cl─La─Cl bending) and 207 cm^−1^ (La─Cl stretching), accompanied by oxygen‐associated vibrations at 330 cm^−1^ (La─O─La bridging) and 432 cm^−1^ (terminal La─O stretching), confirming LaOCl formation.^[^ ^41^ ^]^ The spectral evolution is consistent with the XRD‐detected phase progression, validating La_2_O_3_→LaOCl transformation. The spectrum collected after 770 min of reaction shows two dominant bands at 184 and 206 cm^−1^, indicative of the formation of LaCl_3_ species.^[^ ^33^ ^]^ Residual weak signals at 330/432 cm^−1^ indicate incomplete LaOCl conversion, highlighting kinetic limitations in lattice oxygen substitution.

To elucidate the evolution of surface electronic states during the chlorination reaction, X‐ray photoelectron spectroscopy (XPS) analysis was conducted. The Cl 2p region shown in Figure 2c can be deconvoluted into two peaks at ca. 198.8 and 200.4 eV, which are attributed to spin‐orbit splitting at the 2p_3/2_ and 2p_1/2_, respectively. Since the binding energy of the La 4p_3/2_ electrons overlaps with that of the Cl 2p regions, two additional peaks corresponding to the La 4p_3/2_ (195.1 eV) and a satellite peak (196.5 eV) were also fitted.^[^ ^42^ ^]^ The peak of Cl shifts to a lower binding energy, while the peak of La 4p_3/2_ shifts to a higher binding energy after the reaction. Meanwhile, the peak of La 3d also shifted to higher binding energy (Figure S3). This implies that La atoms donate electrons to Cl atoms. Quantitative analysis of elemental surface composition (Figure 2d) demonstrates a monotonic increase in Cl/La atomic ratio (derived from peak area integration) accompanied by oxygen depletion, confirming progressive replacement of surface oxygen with chlorine adatoms.

Density functional theory (DFT) calculations were performed to map the thermodynamic landscape of surface chlorination (Figure 2f). The Gibbs free energy profile reveals a stepwise chlorination mechanism. Initial exothermic adsorption of HCl on La_2_O_3_ (Δ*G* = −2.08 eV per HCl molecule) precedes saturation‐induced phase transformation to LaOCl, followed by metastable LaOCl decomposition into LaCl_3_ under continuous HCl exposure. Notably, the calculated energy barrier for LaOCl → LaCl_3_ conversion (Δ*G* = −1.82 eV per HCl molecule) corroborates its observed instability in HCl‐rich environments. This synergy between theory and experiment conclusively establishes HCl‐mediated phase evolution as a thermodynamically favored process, governed by sequential surface chlorination.

The catalytic activity of the as‐synthesized La_2_O_3_ was measured through the performance of long‐term experiments under C_2_H_6_ chlorination conditions. As illustrated in Figure 2e, the activity of the La_2_O_3_ catalyst displays a dynamic trend throughout the reaction. In the initial reaction stage, the selectivity for 1,2‐C_2_H_4_Cl_2_ is relatively low, which can be ascribed to the relatively inert surface of La_2_O_3_ that had undergone high‐temperature calcination. With the progression of the reaction, the selectivity for 1,2‐C_2_H_4_Cl_2_ exhibits a continuous increase, as La_2_O_3_ is chlorinated to form LaOCl; which is evidenced by the above time‐resolved spectroscopic and structural characterization. It is well established that LaOCl serves as the active phase for the catalytic chlorination of C_2_H_6_ to 1,2‐C_2_H_4_Cl_2_.^[^ ^30^ ^]^ Nevertheless, as the reaction proceeded further, the structural degradation of LaOCl triggers the over‐chlorination of 1,2‐C_2_H_4_Cl_2_, consequently causing the main product to gradually shift from 1,2‐C_2_H_4_Cl_2_ to C_2_H_3_Cl_3_. Notably, the occurrence of the side reaction of over‐chlorination consumes the Cl_2_ that is fed at stoichiometric ratio, resulting in insufficient Cl_2_ to drive the conversion of C_2_H_6_. Therefore, an inverse correlation between over‐chlorination selectivity and C_2_H_6_ conversion is observed. This shift in product selectivity may be attributed to the structural evolution to LaCl_3_ species and the presence of hydroxyl species on the catalyst surface, both of which can dynamically form during the reaction and influence the reaction pathway. We believe that such structural degradation of LaOCl under Cl_2_/HCl‐rich conditions is a common phenomenon for other rare‐earth oxide catalysts, highlighting the need to identify the primary driving forces behind the over‐chlorination of 1,2‐C_2_H_4_Cl_2_.

### The Role of LaCl_3_ Species on Ethane Chlorination

**Figure 3:**
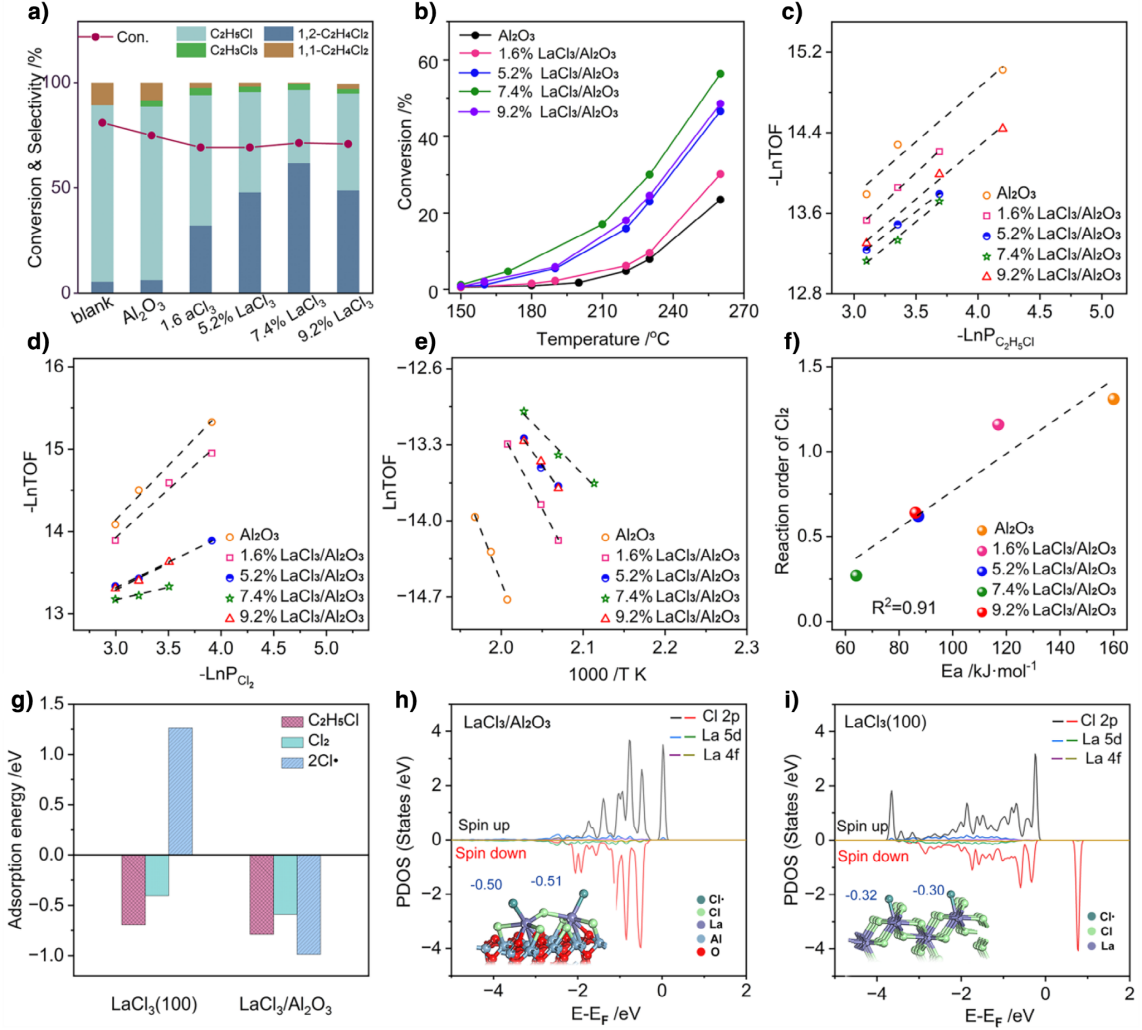
a) Activity tests over empty tube and the LaCl_3_/Al_2_O_3_ catalysts. Reaction conditions: C_2_H_6_/Cl_2_/N_2_ = 4:9:87, 260 °C, WHSV = 2000 mL h^−1^ g^−1^. b) Conversion as a function of temperature in the chlorination of C_2_H_5_Cl over the LaCl_3_/Al_2_O_3_ catalysts. Reaction conditions: C_2_H_5_Cl/Cl_2_/N_2_ = 3.5:5:91.5, 150–260 °C, WHSV = 5500 mL h^−1^ g^−1^. Reaction orders of C_2_H_5_Cl c) and Cl_2_ d). e) Apparent activation energy of C_2_H_5_Cl chlorination. f) Reaction order of Cl_2_ as a function of the apparent activation energy in C_2_H_5_Cl chlorination over the LaCl_3_/Al_2_O_3_ catalysts. Reaction conditions: C_2_H_5_Cl:Cl_2_ = 1.5–4.5:2–5, 200–230 °C, WHSV = 6000–8000 mL h^−1^ g^−1^. g) Adsorption energies of surface species (C_2_H_5_Cl, Cl_2_, and Cl·) on the sites of LaCl_3_/Al_2_O_3_ and LaCl_3_(100). PDOS analysis of Cl· adsorbed on the surface of LaCl_3_/Al_2_O_3_ h) and LaCl_3_(100) i).

As LaCl_3_ is the primary phase present after reaction, we first investigated the mechanistic role of this species in C_2_H_6_ chlorination. To systematically explore this, we employed a model catalyst approach by synthesizing a series of LaCl_3_/θ‐Al_2_O_3_ catalysts with precisely controlled LaCl_3_ loadings (denoted as *x*% LaCl_3_/Al_2_O_3_, where *x* indicates La wt.% loading quantified by ICP‐OES, Table S1). θ‐Al_2_O_3_, which is chemically inert under reaction conditions (as evidenced by control experiments showing comparable activity between θ‐Al_2_O_3_ and the empty reactor, Figure 3a), serves as a stable support for LaCl_3_. XRD characterization (Figure S4) confirmed the evolution of LaCl_3_·3H_2_O crystallinity with increasing loadings. At La loadings below 7.4 wt.%, no discernible diffraction peaks associated with LaCl_3_ phases are observed, indicating atomic‐level dispersion of La species on the θ‐Al_2_O_3_ surface. This conclusion was further corroborated by atomic‐resolution scanning transmission electron microscopy (STEM), which confirmed the absence of La‐containing nanoparticles and demonstrated uniform atomic dispersion of La across the support (Figures S5 and S6). However, as the LaCl_3_ loading increases further, diffraction peaks for LaCl_3_·3H_2_O emerges at 15° in the XRD patterns, indicating that LaCl_3_ species start to aggregate and form nanoparticles.

The structure–activity relationship for the range of model catalysts was elucidated subsequently. Figure 3a shows the conversion of C_2_H_6_ and the product distribution on the LaCl_3_/Al_2_O_3_ catalysts. By analyzing the activity of the catalysts as well as of θ‐Al_2_O_3_, it is noted that over all the catalysts, the C_2_H_6_ conversion is across a rather narrow range from 60% to 66%, indicating virtually no activity difference between the catalysts and θ‐Al_2_O_3_. This is in line with the studies of Olsbye et al., which have shown that C_2_H_6_ chlorination can be feasibly initiated by Cl· via a gas‐phase pathway, which drives virtually exclusive formation of C_2_H_5_Cl (>83.7%) up to 66.6% C_2_H_6_ conversion at 260 °C (Figure S7). Notably, increasing the reaction temperature did not favor the transformation of C_2_H_5_Cl into 1,2‐C_2_H_4_Cl_2_, but instead the undesired 1,1‐C_2_H_4_Cl_2_. Indeed, the gas‐phase Cl· driven chlorination of C_2_H_5_Cl is a major side reaction because chlorination occurs preferentially at the chlorine‐functionalized carbon, hindering the generation of the desired isomer. Interestingly, the incorporation of LaCl_3_ into θ‐Al_2_O_3_ significantly enhances the formation of 1,2‐C_2_H_4_Cl_2_ and exhibits remarkable structural sensitivity. The 1,2‐C_2_H_4_Cl_2_ selectivity shows a marked increase from 32% to 62% with La loading increasing from 1.6% to 7.4%, followed by subsequent inhibition at higher loadings. Mechanistic investigations via the temperature‐programmed surface reaction (TPSR) demonstrate the chemical inertness of lattice chlorine in LaCl_3_ (Figure S8), as evidenced by the absence of 1,2‐C_2_H_4_Cl_2_ formation, which hinders its participation as a Cl donor in the chlorination of C_2_H_5_Cl.

To rationalize the differences observed in catalytic performance, extensive kinetic tests were performed for the reaction of C_2_H_5_Cl with Cl_2_ (Table S2). The light‐off curves of C_2_H_5_Cl chlorination reveal the limitations of θ‐Al_2_O_3_ in catalyzing the chlorination of C_2_H_5_Cl, requiring higher temperatures to initiate the reaction (Figures 3b and S9). However, increasing the temperature mainly induced the formation of 1,1‐C_2_H_4_Cl_2_, indicating that the chlorination of C_2_H_5_Cl on the θ‐Al_2_O_3_ surface is still controlled by the gas‐phase radical pathway (Figure S9a). Interestingly, the loading of LaCl_3_ significantly shifts the light‐off curves to lower temperatures, the extent of which is related to the LaCl_3_ loading, indicating a catalytic effect on C_2_H_5_Cl chlorination. More importantly, the chlorination of C_2_H_5_Cl over all the LaCl_3_/Al_2_O_3_ catalysts predominantly resulted in the formation of 1,2‐C_2_H_4_Cl_2_ (60–100% selectivity). These observations indicate a cascade mechanism for C_2_H_6_ chlorination, where the transformation of C_2_H_6_ into C_2_H_5_Cl is likely dominated by gas‐phase radical pathways, while the role of the catalyst is to activate Cl_2_ and intermediate C_2_H_5_Cl to facilitate the formation of 1,2‐C_2_H_4_Cl_2_.

Temperature‐programmed desorption (TPD) experiments show negligible adsorption for C_2_H_5_Cl for all catalysts (Figure S10), indicating that C_2_H_5_Cl only weakly adsorbs onto the catalyst surface and that the presence of LaCl_3_ does not significantly enhance this adsorption. This suggests that the mechanism by which LaCl_3_ enhances 1,2‐C_2_H_4_Cl_2_ selectivity involves alternative pathways that do not significantly rely on the adsorption and activation of C_2_H_5_Cl on the catalyst surface. This inference is consistent with kinetic findings, which reveals near‐unity reaction orders for C_2_H_5_Cl independent of LaCl_3_ loadings (Figure 3c). In contrast, the reaction order of Cl_2_ exhibits a tendency to decrease first and then increase as the loading of LaCl_3_ increases (Figure 3d). Notably, the reaction order of Cl_2_ decreases considerably over the 7.4% LaCl_3_/Al_2_O_3_ catalyst, with the reaction order being only 0.3. Interestingly, a negative correlation is found between the reaction order of Cl_2_ and the 1,2‐C_2_H_4_Cl_2_ selectivity (Figure S11). These findings clearly demonstrate that the rate‐determining step involving the Cl radical is significantly structure‐sensitive, with highly dispersed LaCl_3_ playing a promotional role, and this effect is quenched when LaCl_3_ agglomerates. This can be rationalized by the relationship in Figure 3f, which evidences a linear correlation between the apparent activation energy and the reaction order of Cl_2_. Arrhenius analysis reveals a loading‐dependent activation energy of C_2_H_5_Cl chlorination in the range of 64.0–117.4 kJ mol^−1^ (Figure 3e). The activation energy is significantly lower for the highly dispersed LaCl_3_ species, while the aggregated LaCl_3_ particles formed at higher loadings result in a higher activation energy.

To gain atomic‐level insights into the structural sensitivity of C_2_H_5_Cl chlorination at LaCl_3_ sites, DFT calculations were performed using two model systems: (i) a (LaCl_3_)_2_ dimer supported on θ‐Al_2_O_3_ (110) to mimic highly dispersed LaCl_3_ (denoted as LaCl_3_/Al_2_O_3_), and (ii) chlorine‐terminated LaCl_3_(100) to represent aggregated configurations. Notably, the interaction between La atoms in the (LaCl_3_)_2_ dimer and surface oxygen atoms significantly weakens the La─Cl bond. Upon structural optimization, one La─Cl bond within the dimer undergoes cleavage, resulting in a free Cl ion that is captured by an adjacent Al^3+^ site. The resulting dimer of La ions is subsequently anchored on the Al_2_O_3_ surface by hybrid O/Cl coordination. This is consistent with the study by Chen et al., which has shown that CuCl_2_ undergoes dissociative adsorption on an γ‐Al_2_O_3_ (110) surface, where only one chloride ion binds to copper, and the other binds to the Al_2_O_3_ surface.^[^ ^43^ ^]^

Typically, interactions between rare‐earth metal sites and molecules primarily involve electron orbitals below the Fermi level, as these electrons dominate bonding and antibonding interactions at the metal surface.^[^ ^44^, ^45^ ^]^ Projected density of states (PDOS) analysis demonstrates negligible perturbation to La 4f and 5d orbitals below the Fermi level in the (LaCl_3_)_2_ dimer (Figure S12). This finding explains the structural insensitivity observed in the adsorption of C_2_H_5_Cl and Cl_2_ at La sites, with both exhibiting weak adsorption energies (Figure 3g). These findings align with kinetic data showing C_2_H_5_Cl reaction orders (0.94–1.1) independent of LaCl_3_ dispersion. Furthermore, it is important to note that the activation of Cl_2_ is not a critical factor in C_2_H_6_ chlorination, as the thermal scission of Cl_2_ into two Cl radicals occurs readily even at 200 °C (Figure S7).

Interestingly, the bonding of the (LaCl_3_)_2_ dimer with interface oxygen significantly enhances the spin degenerate empty states above the Fermi level, which involve the atomic orbitals of La 4f and O 2p (Figure S12). These vacant orbitals can act as Lewis acid sites, facilitating electron donation from Cl· via frontier orbital interactions.^[^ ^46^ ^]^ The enhanced electron‐accepting capacity of La centers strengthens their interaction with Cl· through charge transfer. Notably, Cl· retains a significant amount of electron density at the Fermi level when interacting with the (LaCl_3_)_2_ dimer (Figure 3h). These highly active electrons potentially lower the activation energy for subsequent chlorination reactions. In contrast, on the LaCl_3_(100) surface, Cl· exhibits weak interactions, resulting in Cl 2p electrons occupying states above the Fermi level (Figure 3i). This conclusion is further confirmed by the calculated adsorption energies (Δ*E*; the more negative the value, the stronger the binding), where the value for Cl· adsorption on the (LaCl_3_)_2_ dimer (−0.98 eV) is much less than that on LaCl_3_(100) (1.26 eV) (Figure 3g). The repulsion of the Cl· on the LaCl_3_(100) surface hinders further chlorination of C_2_H_5_Cl, making the product of C_2_H_6_ chlorination primarily C_2_H_5_Cl. The observed performance degradation at aggregated LaCl_3_ highlights the necessity of controlling the degree of chlorination of the catalyst to prevent crystalline phase segregation.

**Figure 4:**
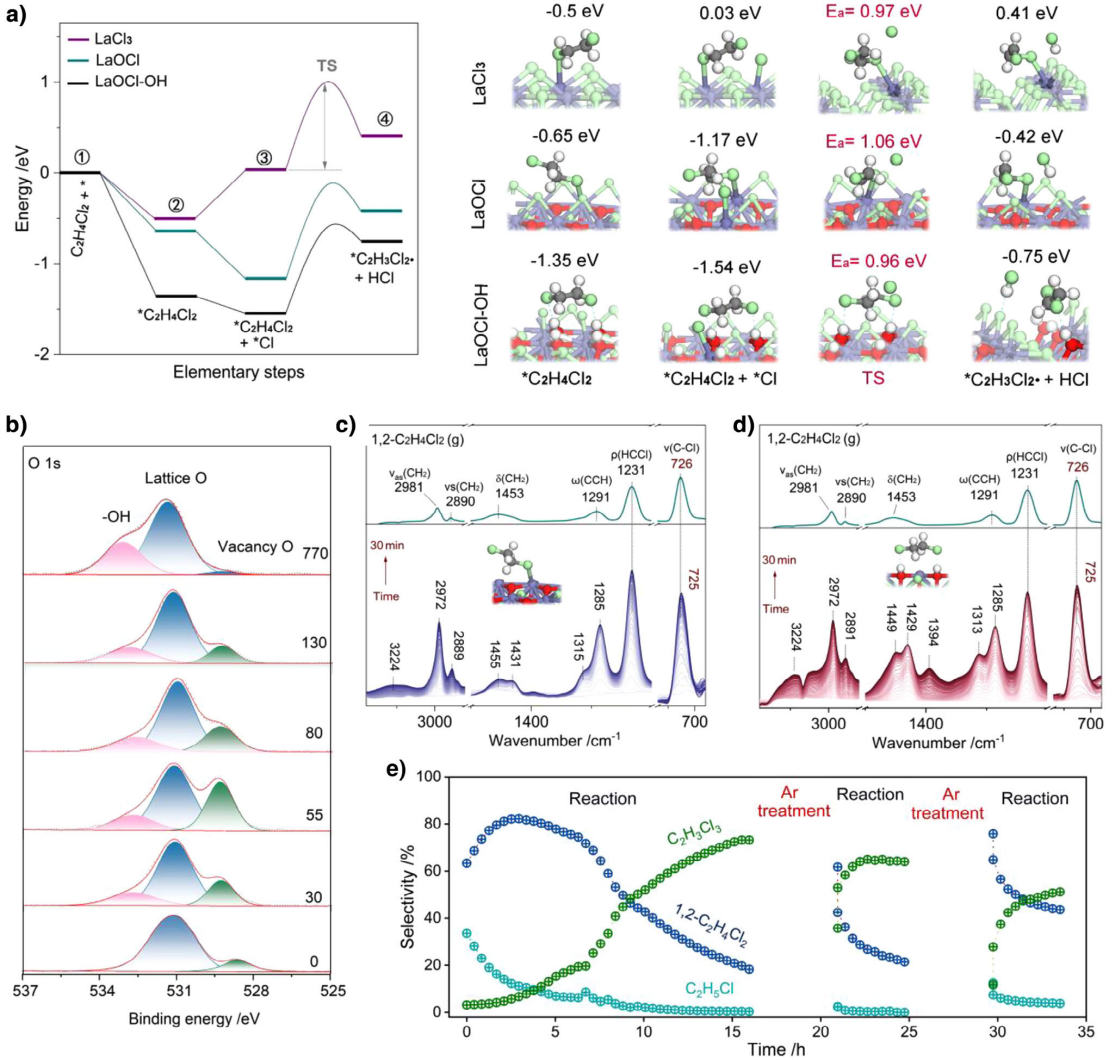
Determining the role of hydroxyl groups in C_2_H_6_ chlorination. a) Energy profile for 1,2‐C_2_H_4_Cl_2_ chlorination on the surfaces of LaCl_3_, LaOCl, and LaOCl‐OH. The numbers above the models indicate the adsorption energies of different adsorption states. b) O 1s XPS spectra of La_2_O_3_ recorded at different reaction times (unit: minute). In situ DRIFTS of 1,2‐C_2_H_4_Cl_2_ adsorption on La catalysts sampled at 55 min c) and 770 min d). e) Activity profile of the La_2_O_3_ catalyst at 260 °C. The catalyst underwent periodic Ar treatment at 350 °C for 5 h after 15 h of reaction, followed by re‐exposure to reaction conditions (C_2_H_6_/Cl_2_/N_2_ = 4:9:87, 260 °C, WHSV = 2000 mL h^−1^ g^−1^).

To elucidate the atomic‐level mechanism by which phase transformation governs over‐chlorination, we simulated the adsorption of 1,2‐C_2_H_4_Cl_2_ on the LaOCl (100) surface (Figure 4a). To model realistic reaction conditions, unsaturated La sites on the LaOCl surface were saturated with Cl radicals, as thermal dissociation of Cl_2_ at 260 °C readily generates gas‐phase Cl radicals (Figure S13). The calculations reveal weak adsorption of 1,2‐C_2_H_4_Cl_2_ on the Cl‐saturated LaOCl surface, with an adsorption energy of −0.65 eV. Crucially, the energy barrier for Cl radical‐mediated H abstraction from adsorbed 1,2‐C_2_H_4_Cl_2_ to form the 1,2‐C_2_H_3_Cl_2_⋅ radical (1.06 eV) remains significantly higher than the adsorption energy. This thermodynamic preference for desorption over further chlorination aligns with experimental observations of high 1,2‐C_2_H_4_Cl_2_ selectivity on LaOCl. To probe the role of LaCl_3_ in over‐chlorination, we subsequently examined 1,2‐C_2_H_4_Cl_2_ adsorption on the LaCl_3_(100) surface (Figure 4a). Similarly, weak linear adsorption (−0.5 eV) was observed, with the adsorption energy being significantly lower than the energy barrier (0.97 eV) for subsequent chlorination. This confirms that LaCl_3_ formation alone does not drive over‐chlorination.

### Hydroxyl‐Mediated Over‐Chlorination

As illustrated by the DFT calculations in Figure 2f, the exothermic adsorption of HCl on LaOCl surfaces drives a thermodynamically favorable chlorination process, ultimately saturating La sites with Cl atoms and hydroxylating surface oxygen species. Time‐resolved O 1s XPS spectra provide direct evidence of this dynamic surface hydroxylation (Figure 4b). After treatment at 800 °C, hydroxyl groups on La_2_O_3_ surfaces are completely removed, leaving only lattice oxygen and a minor concentration of oxygen vacancies, which appear at 531.2 and 528.6 eV, respectively.^[^ ^47^ ^]^ However, under Cl_2_/HCl atmospheres, the La_2_O_3_ surface is rapidly etched, leading to a significant increase in the number of oxygen vacancy species, accompanied by the generation of hydroxyl species, identified by the peak at 532.7 eV.^[^ ^48^ ^]^ As the reaction progresses, water produced during chlorination progressively quenches oxygen vacancies, while a substantial amount of hydroxyl groups is generated. Notably, due to the kinetic limitations in the complete chlorination of LaOCl to LaCl_3_, a minor concentration of oxygen vacancies remains on the catalyst surface after 770 min of reaction. Fourier transform infrared (FTIR) spectroscopy experiments further indicate the hydroxyl groups accumulation, with a distinct peak corresponding to hydroxyl groups observed at 3385 cm^−1^ after the reaction (Figure S14).^[^ ^49^ ^]^

Subsequently, we focus on the role of surface hydroxyls in the adsorption of 1,2‐C_2_H_4_Cl_2_. To obtain direct evidence of hydroxyl group involvement in C─Cl bond activation, we performed in situ diffuse reflectance infrared Fourier transform spectroscopy (DRIFTS) studies to investigate the adsorption of 1,2‐C_2_H_4_Cl_2_ on La catalysts sampled at 55 min (LaOCl‐dominated phase) and 770 min (denoted as LaOCl‐OH) under reaction conditions. Dynamic analysis of surface‐adsorbed species demonstrates well‐defined chemisorption states of 1,2‐C_2_H_4_Cl_2_ on both catalysts at room temperature. As illustrated in Figure 4c, the infrared spectra display distinct vibrational bands at 2971 cm^−1^ (*ν*_as_(CH_2_) asymmetric stretching), 2890 cm^−1^ (*ν*_s_(CH_2_) symmetric stretching), 1455 cm^−1^ (*δ*(CH_2_) scissoring), 1289 cm^−1^ (*ω*(CCH) wagging), 1232 cm^−1^ (*ρ*(HCCl) rocking), and 725 cm^−1^ (*ν*(C─Cl) stretching).^[^ ^50^, ^51^ ^]^ Notably, these adsorbed species exhibit systematic blueshifts compared to the intrinsic vibrational frequencies of gaseous 1,2‐C_2_H_4_Cl_2_. Furthermore, weak splitting of the *δ*(CH_2_) mode at 1453 cm⁻¹ and the *ω*(CCH) mode at 1291 cm⁻¹ are observed in the adsorbed state, indicative of symmetry disruption and electron density redistribution during chemisorption.

Intriguingly, on the LaOCl‐OH surface (Figure 4d), the *δ*(CH_2_) and *ω*(CCH) peaks exhibit enhanced splitting, accompanied by the emergence of a new C─H bending vibration at 1394 cm^−1^. These spectral changes suggest a transition from linear to bridged adsorption geometry for 1,2‐C_2_H_4_Cl_2_, further perturbing molecular symmetry and amplifying vibrational coupling of C─H modes.^[^ ^52^ ^]^ This conclusion is strongly supported by a broadened absorption band at 3224 cm^−1^, assigned to O─H⋯Cl hydrogen bonding interactions between surface μ‐OH groups and adsorbed molecules.^[^ ^51^ ^]^ These findings provide unequivocal evidence for hydroxyl‐mediated adsorption of 1,2‐C_2_H_4_Cl_2_ on LaOCl surfaces, as corroborated by PDOS analysis, which reveals hydrogen‐bonding interactions between surface hydroxyls and the Cl atoms of 1,2‐C_2_H_4_Cl_2_ (Figure S15). We thus constructed a hydroxyl‐covered LaOCl model (LaOCl‐OH). Intriguingly, 1,2‐C_2_H_4_Cl_2_ adopts a bidentate adsorption mode via hydrogen bonding with surface hydroxyls, dramatically increasing the adsorption energy to −1.35 eV. The enhanced adsorption enables 1,2‐C_2_H_4_Cl_2_ to overcome the chlorination barrier (0.96 eV), identifying hydroxyl‐induced adsorption as the primary driving force of over‐chlorination.

To further validate the hydrogen bonding as the driving force behind the over‐chlorination of 1,2‐C_2_H_4_Cl_2_, we subjected the spent catalyst to treatment with Ar for 5 h (Figure 4e). FTIR experiments indicate that treatment at 350 °C effectively reduces the number of hydroxyl groups on the catalyst surface (Figure S14). Interestingly, after Ar treatment, the selectivity of the catalyst for 1,2‐C_2_H_4_Cl_2_ recovers from 18.3% to 61.7% (Figure 4e), while the selectivity for C_2_H_3_Cl_3_ is suppressed from 73.2% to 35.8%. Notably, as the reaction continues, surface hydroxyls reaccumulate, leading to a shift in chlorination products from 1,2‐C_2_H_4_Cl_2_ to C_2_H_3_Cl_3_. It needs to be emphasized that repeated Ar treatments only induce the dynamic reversible changes of surface hydroxyl sites, with almost no impact on La sites. The above results fully demonstrate that the hydroxyl groups on the catalyst surface induce the over‐adsorption of 1,2‐C_2_H_4_Cl_2_, which is the intrinsic driving force for over‐chlorination.

## Conclusions

In this study, we provide a comprehensive understanding of the chlorination of C_2_H_6_ over lanthanum‐based catalysts. By employing advanced spectroscopic techniques and DFT calculations, we reveal the structural evolution from La_2_O_3_ to LaOCl and subsequently to LaCl_3_ under HCl/Cl_2_‐rich conditions. This transformation significantly influences the product distribution, shifting selectivity from C_2_H_5_Cl to 1,2‐C_2_H_4_Cl_2_, and ultimately to C_2_H_3_Cl_3_. Our research elucidates the pivotal roles played by LaCl_3_ species and surface hydroxyl groups in the chlorination process. Monolayer‐dispersed LaCl_3_ clusters, stabilized by La─O interactions with the support, effectively stabilize Cl·, thereby enhancing the selectivity toward 1,2‐C_2_H_4_Cl_2_. In contrast, the aggregated LaCl_3_ nanoparticles repulse Cl· access. Furthermore, we have identified that hydroxyl groups formed during surface chlorination crucially drive the over‐chlorination of 1,2‐C_2_H_4_Cl_2_ through promoting hydrogen‐bond‐assisted bidentate adsorption. The findings of this study are significant as they confirm the structure sensitivity of lanthanum‐catalyzed chlorination and highlight the importance of maintaining metastable LaOCl species and suppressing hydroxyl accumulation to enhance selectivity. Importantly, the unique role of highly dispersed LaCl_3_ in stabilizing Cl· highlights the potential application of lanthanum‐based single‐atom catalysts in C_2_H_6_ chlorination. These insights pave the way for the development of more efficient and selective catalysts for C_2_H_6_ chlorination, potentially leading to breakthroughs in the sustainable production of PVC and other chlorinated hydrocarbons from new renewable resources.

## Experimental Section

For the experimental details, see Supporting Information.

## Conflict of Interests

The authors declare no conflict of interest.

## Supporting information



Supporting Information

## Data Availability

The data that support the findings of this study are available in the Supporting Information of this article.
