# Supplementary material for: Dynamic Active Site Evolution in Lanthanum‐Based Catalysts Dictates Ethane Chlorination Pathways
Source: Angew Chem Int Ed Engl. 2025 Jun 26;64(34):e202505846. doi: 10.1002/anie.202505846 (PMC12363613; doi:10.1002/anie.202505846)
Supplement: Supplementary file 1 — Supporting Information [file ANIE-64-e202505846-s001.docx]

**Supporting information**

**Dynamic Active Site Evolution in Lanthanum-Based Catalysts Dictates Ethane Chlorination Pathways**

Yuting Li^[a,b]+^, Haifeng Qi^[c]+^, Zihan Zhu^[a,b]^, Xia Wu^[a,d]^, Nicholas F. Dummer^[c]^, Stuart H. Taylor^[c]^, Lei Ma^[a]^, Xiaofeng Yang^[a]^, Qinggang Liu*^[a]^, Graham J. Hutchings*^[c]^, and Yanqiang Huang*^[a]^

^[a]^ State Key Laboratory of Catalysis, Dalian Institute of Chemical Physics, Chinese Academy of Sciences, Dalian 116023, China.

^[b]^ University of Chinese Academy of Sciences, Beijing 100049, China.

^[c]^ Max Planck-Cardiff Centre on the Fundamentals of Heterogeneous Catalysis FUNCAT, Cardiff Catalysis Institute, Translational Research Hub, Cardiff University, Maindy Road, Cardiff CF24 4HQ, UK.

^[d]^ Chemical Engineering and Resource Utilization, Northeast Forestry University, Harbin 150040, China.

^+^These authors contributed equally

E-mail: [liuqg@dicp.ac.cn](mailto:liuqg@dicp.ac.cn); [hutch@cardiff.ac.uk](mailto:hutch@cardiff.ac.uk); yqhuang@dicp.ac.cn

**Experimental Section**

**Chemicals and Materials**

Lanthanum nitrate hexahydrate (La(NO_3_)_3_·6H_2_O) and Lanthanum chloride heptahydrate (LaCl_3_·7H_2_O) and Aluminum nitrate nonahydrate (Al(NO_3_)_3_·9H_2_O) were purchased from Macklin Chemical Reagent Factory. Ammonia (NH_3_·H_2_O) was purchased from Tianjin Damao Chemical Reagent Factory.

**Synthesis of** **La_2_O_3_ Catalyst**
La_2_O_3_ was synthesized via an ammonia precipitation method. Briefly, 5.6 g of La(NO_3_)_3_·6H_2_O was dissolved in 150 mL of deionized water under magnetic stirring to form a homogeneous solution. Separately, 25 mL of aqueous ammonia (25% v/v NH_3_·H_2_O) was diluted in 150 mL of deionized water. The alkaline ammonia solution was then added dropwise into the La(NO_3_)_3_ solution under vigorous stirring, resulting in the formation of a white precipitate. The suspension was aged for 8 h at room temperature, followed by centrifugation (8000 rpm, 10 min), thorough washing with deionized water, and drying at 80 °C for 12 h. The dried precursor was calcined in a muffle furnace at 800 °C for 3 h (heating rate: 5 °C·min^-1^) to obtain crystalline La_2_O_3_.

**Synthesis of θ-Al_2_O_3_ Support**
The Al_2_O_3_ support was prepared using a similar ammonia precipitation method. Specifically, 2.0 g of Al(NO_3_)_3_·9H_2_O was dissolved in 150 mL of deionized water under continuous stirring. Aqueous ammonia (10 mL, 25% v/v) was gradually added to the solution, inducing the formation of a white Al(OH)_3_ precipitate. The mixture was aged for 2 h, centrifuged, washed, and dried under identical conditions as described above. The dried powder was calcined at 900 °C for 3 h to yield θ-Al_2_O_3_.

**Preparation of LaCl_3_/Al_2_O_3_ Catalysts**
LaCl_3_/Al_2_O_3_ catalysts were fabricated via incipient wetness impregnation. Aqueous solutions containing varying amounts of LaCl_3_·7H_2_O (to achieve target La loadings) were prepared in 5 mL of deionized water. The θ-Al_2_O_3_ support (1.0 g) was impregnated with the LaCl_3_ solution in five sequential steps, with intermediate drying at 100 °C for 1 h after each step to ensure uniform dispersion. The resulting material was dried at 150 °C for 4 h in air and stored in a desiccator prior to use. La loadings were precisely controlled by adjusting the initial LaCl_3_·7H_2_O concentration.

**Characterization.**

High-resolution transmission electron microscopy (HRTEM) images and energy-dispersive X-ray spectroscopy (EDS) were acquired on a JEM2100F microscope. The microscope was operated at an accelerating voltage of 200 kV. Powder X-ray Diffraction (XRD) Patterns were recorded using a PW3040/60 X'Pert ProSuper (PANalytical) diffractometer equipped with a Cu Kα radiation source operating at 40 kV, 40 mA. X-ray photoelectron spectroscopy (XPS) was measured on a Thermofisher ESCALAB 250Xi instrument, which applies monochromatic Al Kα radiation (hυ = 1486.6 eV) as the X-ray source. Inductively coupled plasma optical emission spectrometer (ICP-OES) was used to measure the content of La in the samples. The Raman spectroscopy was measured on a Nano Wizard instrument with an excitation laser of 532 nm. The hydroxyl groups (OH) on the surface of the catalyst were measured using an INVENIO S infrared spectrometer and the samples were placed in an oven and dried at 155 °C. Temperature-programmed desorption of C_2_H_5_Cl (C_2_H_5_Cl-TPD) was conducted using Micromeritics AutoChem II 2920 chemisorption equipment. Before the measurement, the sample was dried in He at 250 ºC for 150 min, and then cooled to 40 °C for adsorbing C_2_H_5_Cl for 60 min. Subsequently, the gas was switched to He for purging, and the temperature was programmed to increase at a rate of 10 ºC per minute until reaching 400 ºC. A Thermal Conductivity Detector was used to record the signal. Temperature-programmed surface reaction of C_2_H_5_Cl (C_2_H_5_Cl-TPSR) was conducted on a Micromeritics AutoChem II 2920 instrument. Before making the measurement, the sample was dried in He at 250 ºC for 60 min and then cooled to 40 °C. Subsequently, a mixture of 5 vol% C_2_H_5_Cl/N₂ was introduced, and the temperature was increased to 300 ºC at a rate of 10 ºC·min^-1^. Data were recorded by measuring the effluent components using mass spectrometry. *In situ* DRIFTS analysis of the catalyst during 1,2-C_2_H_4_Cl_2_ adsorption were carried out using an Equinox 55 infrared spectrometer (Bruker) equipped with a high-temperature DRIFTS reactor cell and liquid nitrogen-cooled DLATGS detector. All spectra were obtained with a resolution of 4 cm^-1^ and an accumulation of 32 scans. Prior to the 1,2-C_2_H_4_Cl_2_ adsorption test, catalyst samples were treated at 150 °C for 1 h under Ar. Then the cell was cooled to room temperature in pure Ar. The spectra of 1,2-C_2_H_4_Cl_2_ adsorption were recorded after 1,2-C_2_H_4_Cl_2_ was introduced by Ar at 25 ºC.

**Catalyst Evaluation.**

C_2_H_6_ chlorination was conducted at atmospheric pressure in a self-made continuous-flow fixed-bed reactor. The gases C_2_H_6_ (8% in N_2_), C_2_H_5_Cl (9% in N_2_), Cl_2_ (18% in N_2_), Ar (carrier gas), and N_2_ (carrier gas) were fed at controlled flow rates using digital mass-flow controllers (Bronkhorst®) to a mixing unit. The performance of the catalyst was investigated in a fixed-bed quartz reactor (inner diameter 8 mm) with an inlet feed gas composition of C_2_H_6_/Cl_2_/N_2_ = 4:9:87, the reaction temperature was 260 °C, the total flow rate of the gas as 16.7 ml·min^-1^ with 0.5 g of catalyst to give a WHSV = 2000 ml·h^-1^·g^-1^. The gas chromatograph (GC) equipped with a FID detector was used for online analysis of the feed gas and the reaction products.

Kinetic testing of LaCl_3_/Al_2_O_3_ catalysts was carried out in a fixed-bed quartz reactor (8 mm inner diameter). 0.2 g of catalyst was loaded in the reactor and pretreated with a feed gas atmosphere (C_2_H_6_/Cl_2_/N_2_ = 4:9:87) at 260 °C for 6 h under atmospheric pressure. Then, the reaction was carried out with the bed temperature ranging from 140 °C to 260 °C with a feed composition of C_2_H_5_Cl:Cl_2_ = 1.5-4.5:2-5, the total flow rate of the gas was 20-26.7 ml·min^-1^, and the catalyst loading was 0.2 g (WHSV = 6000-8000 ml·h^-1^·g^-1^). For each experimental point, measurements were taken every 20 min, and the average of three or more measurements was used to calculate concentrations.

C_2_H_6_ and C_2_H_5_Cl conversions were calculated from the following equation

$$\text{Con}_{\text{C}_{2}\text{H}_{6}(\text{C}_{2}\text{H}_{5}\text{Cl})}=\frac{\text{C}_{2}\text{H}_{6}(\text{C}_{2}\text{H}_{5}\text{Cl})_{\text{inlet}}-\text{C}_{2}\text{H}_{6}(\text{C}_{2}\text{H}_{5}\text{Cl})_{\text{outlet}}}{\text{C}_{2}\text{H}_{6}(\text{C}_{2}\text{H}_{5}\text{Cl})_{\text{inlet}}}$$

The product selectivity *X* (*x*= C_2_H_4_, C_2_H_5_Cl, C_2_H_3_Cl, 1,2-C_2_H_4_Cl_2_, 1,1-C_2_H_4_Cl_2_, and 1,1,2-C_2_H_3_Cl_3_ selectivity) was calculated according to the following equation

$$\text{Sel}_{\text{x}}=\frac{\text{X}_{\text{outlet}}}{\text{C}_{2}\text{H}_{6\text{（inlet）}}-\text{C}_{2}\text{H}_{6\text{（outlet）}}}$$

where "inlet" and "outlet" represent chemicals in the inlet and outlet, separately.

**Density Functional Theory Calculations**

All calculations were performed with periodic boundary conditions at the density functional theory level, implemented in the Vienna ab initio simulation package (VASP, version 5.4.4)^[1-3]^, using the Perdew-Burke-Ernzerhof (PBE) functional^[4]^. Projector-augmented wave (PAW) pseudopotentials were used to consider the inner electrons^[5]^, whereas valence electronic states were expanded in plane waves with an energy cutoff of 450 eV. Van der Waals contributions were included by using the D3 dispersion correction method. The criteria for electronic and geometry optimization convergence were set to 10^-5^ eV and 0.02 eV/Å ^[6]^, respectively. The transition states were obtained using the climbing image nudged elastic band (CI-NEB) method by relaxing the force below 0.05 eV/Å. Reciprocal-space integration over the Brillouin zone was approximated through k-point grid using 2×2×1.To elucidate the surface chlorination behavior of La-based catalysts, we employed three distinct models: regular O-terminated La_2_O_3_ (001), chlorine-terminated LaOCl (100) and HCl-terminated LaOCl (100). The La_2_O_3_ (001) was based on a 2×2 periodic unit cell consisting of 6 layers, while the LaOCl (100) was constructed using a 3×2 periodic unit cell with 4 layers. To mimic highly dispersed LaCl_3_, a (LaCl_3_)_2_ dimer supported on θ-Al_2_O_3_ (110) was employed (LaCl_3_/Al_2_O_3_). In contrast, chlorine-terminated LaCl_3_(100) was used as the model based on a 3×2 periodic unit cell with 7 layers to represent aggregated configurations. The bottom layers (3 for La_2_O_3_, 2 for LaOCl, 2 for LaCl_3_/Al_2_O_3_ and 3 for LaCl_3_) were constrained during the calculations to simulate bulk properties, and the remaining top layers were optimized with an adsorbate. A vacuum spacing of 1.5 nm was used in all surface models. Chlorination of the surface was considered in the uppermost layer by replacement of O atoms with Cl and H atoms. All adsorption and surface reaction energies were calculated at 0 K without zero-energy corrections. The adsorption energy was calculated according to *E*_ads_= *E_X_*_/slab_− [*E*_slab_+ *E_X_*], where *E_X_*_/slab_ is the total energy of the slab with adsorbates in its equilibrium geometry, *E*_slab_ is the total energy of the bare slab, and *E_X_* is the total energy of the free adsorbates in the gas phase. Therefore, the more negative the *E*_ads_, the stronger the adsorption.


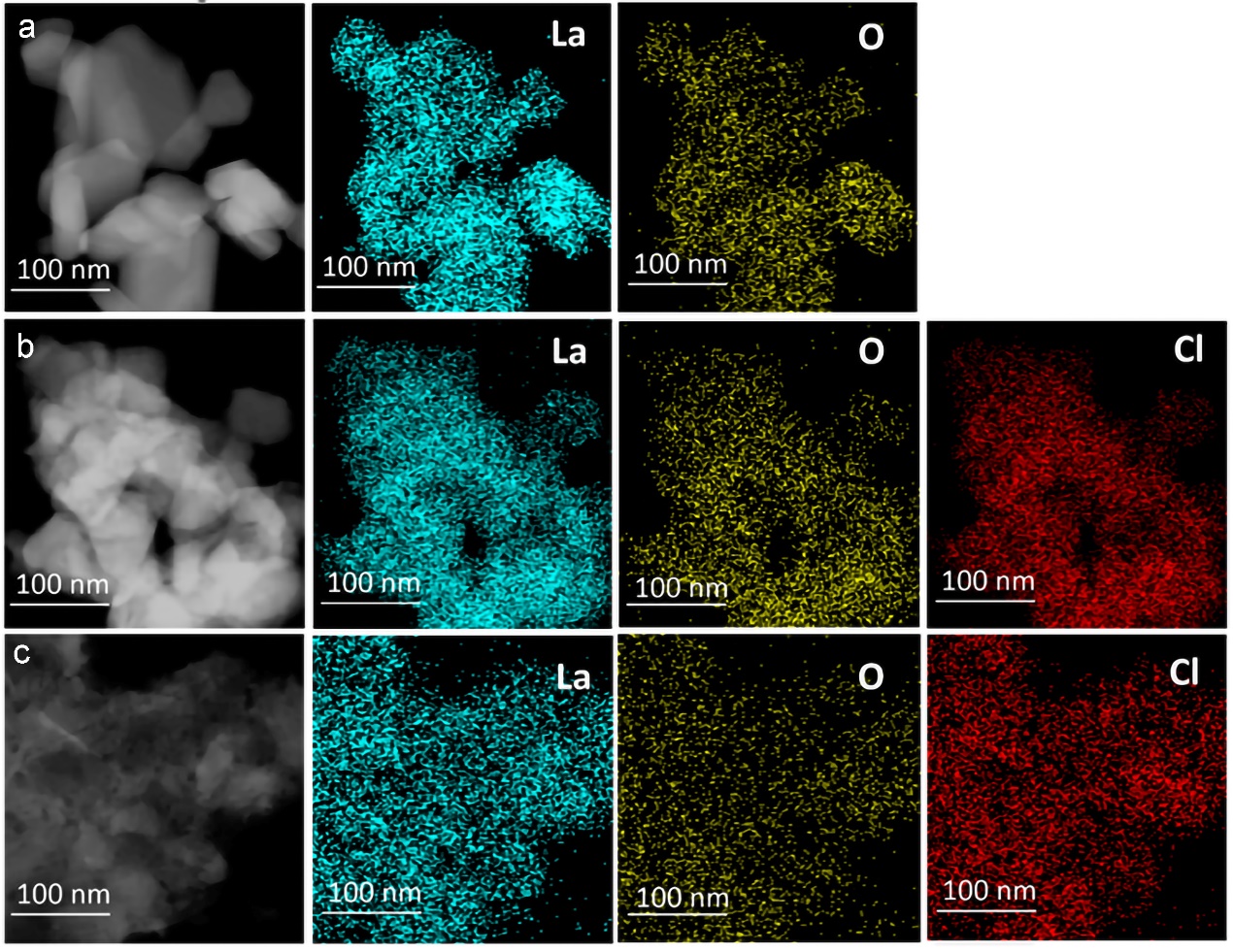


**Figure S1.** HRTEM images and elemental maps (La, O, Cl) of La_2_O_3_ catalysts sampled at0 min (**a**), 55 min (**b**) and 770 min (**c**) of reaction.


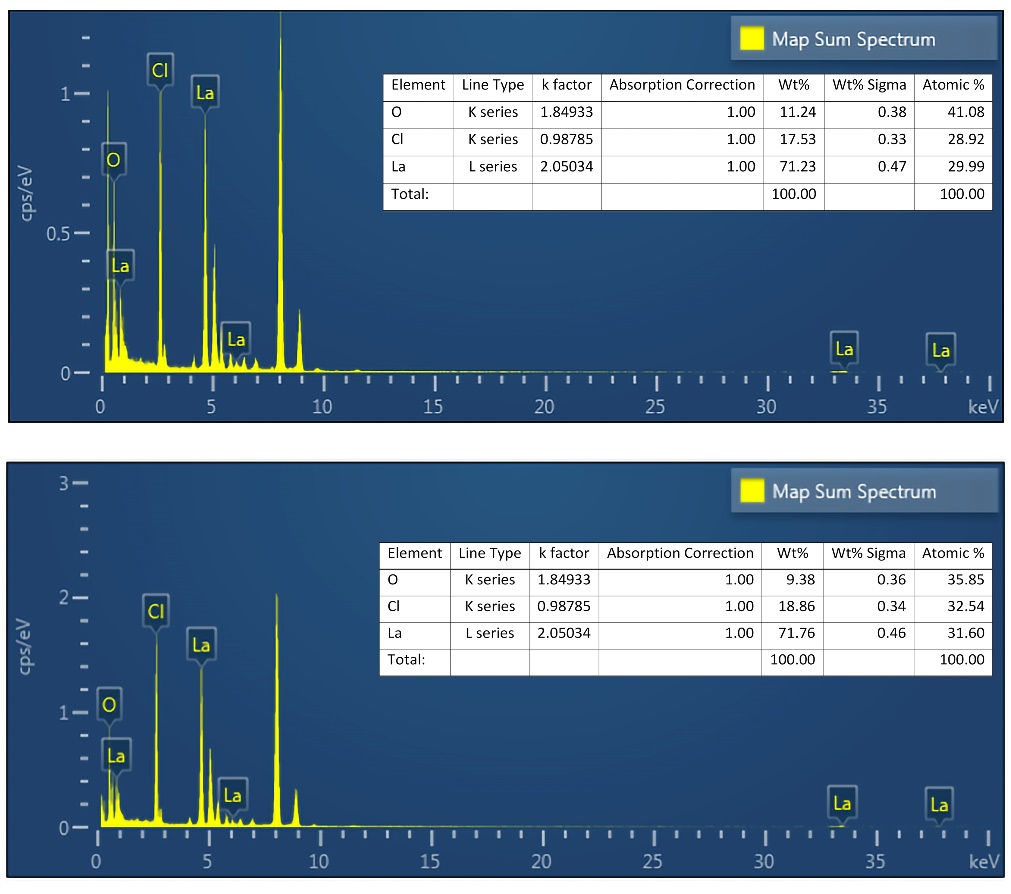


**Figure S2.** EDS spectra and corresponding elemental analysis of the La_2_O_3_ catalyst after 770 min of reaction.


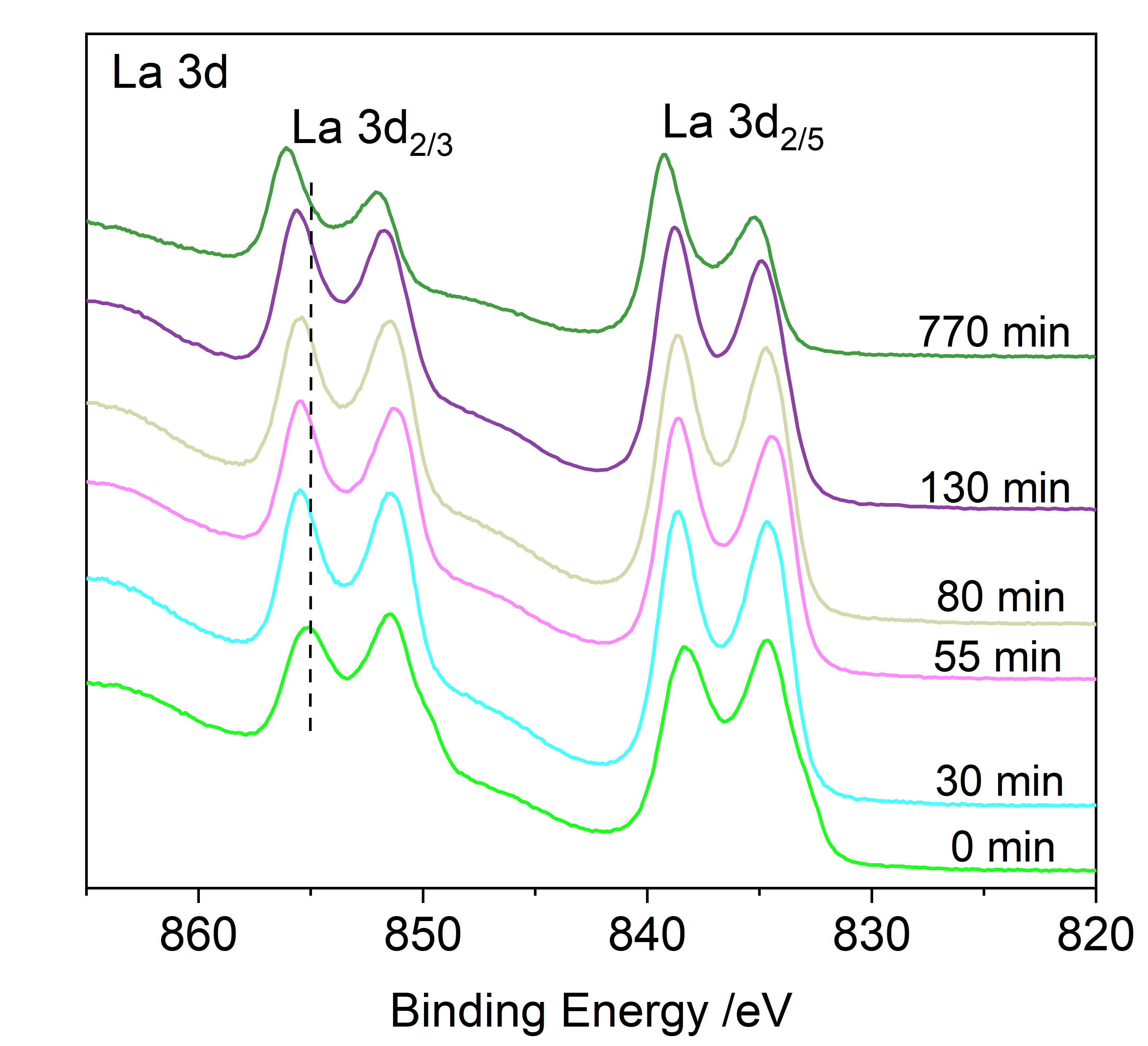


**Figure S3.** La 3d XPS spectra of the La_2_O_3_ catalysts sampled at different reaction times.


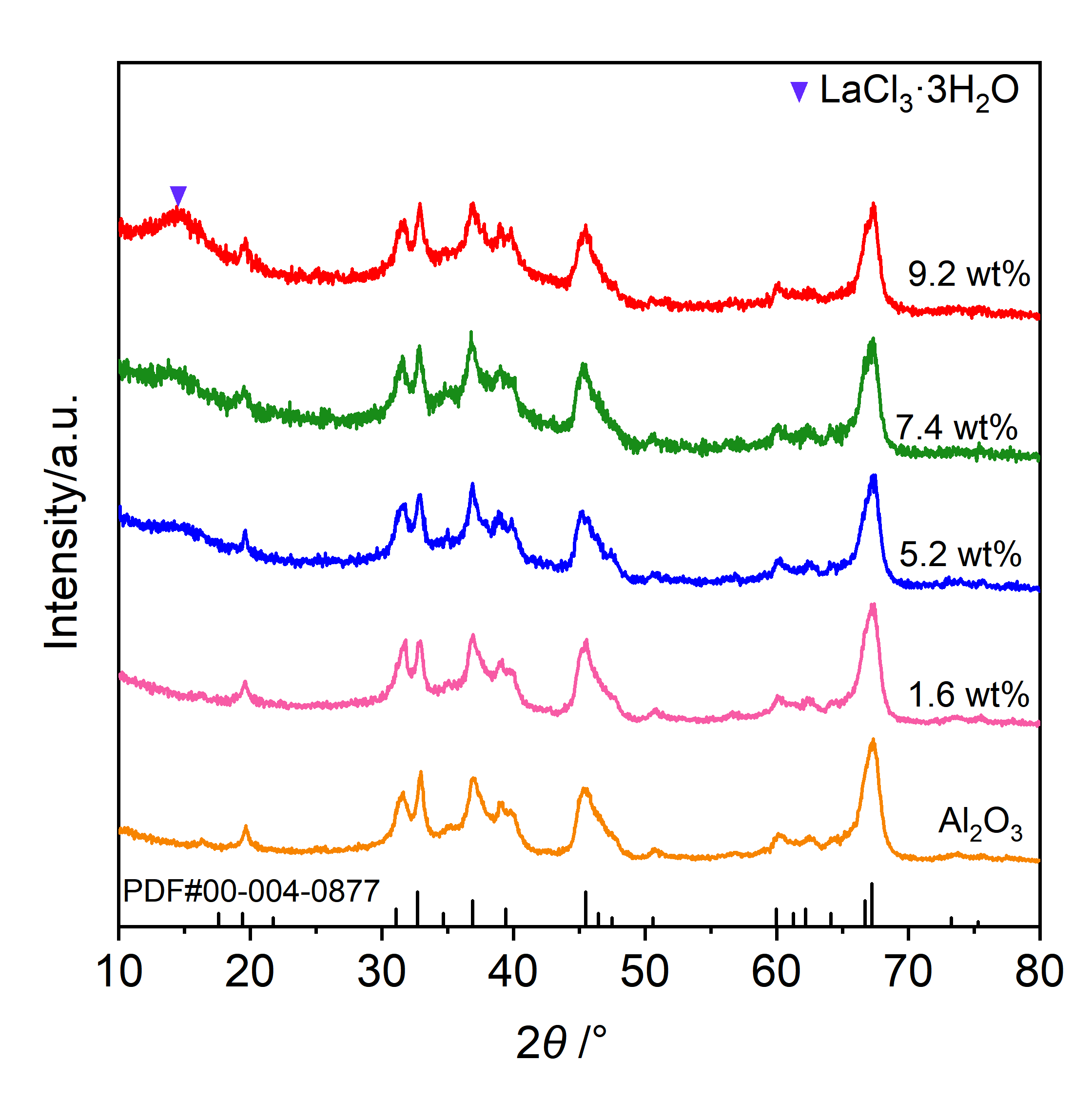


**Figure S4.** XRD patterns of the θ-Al_2_O_3_ and LaCl_3_/Al_2_O_3_ catalysts with different La loadings.


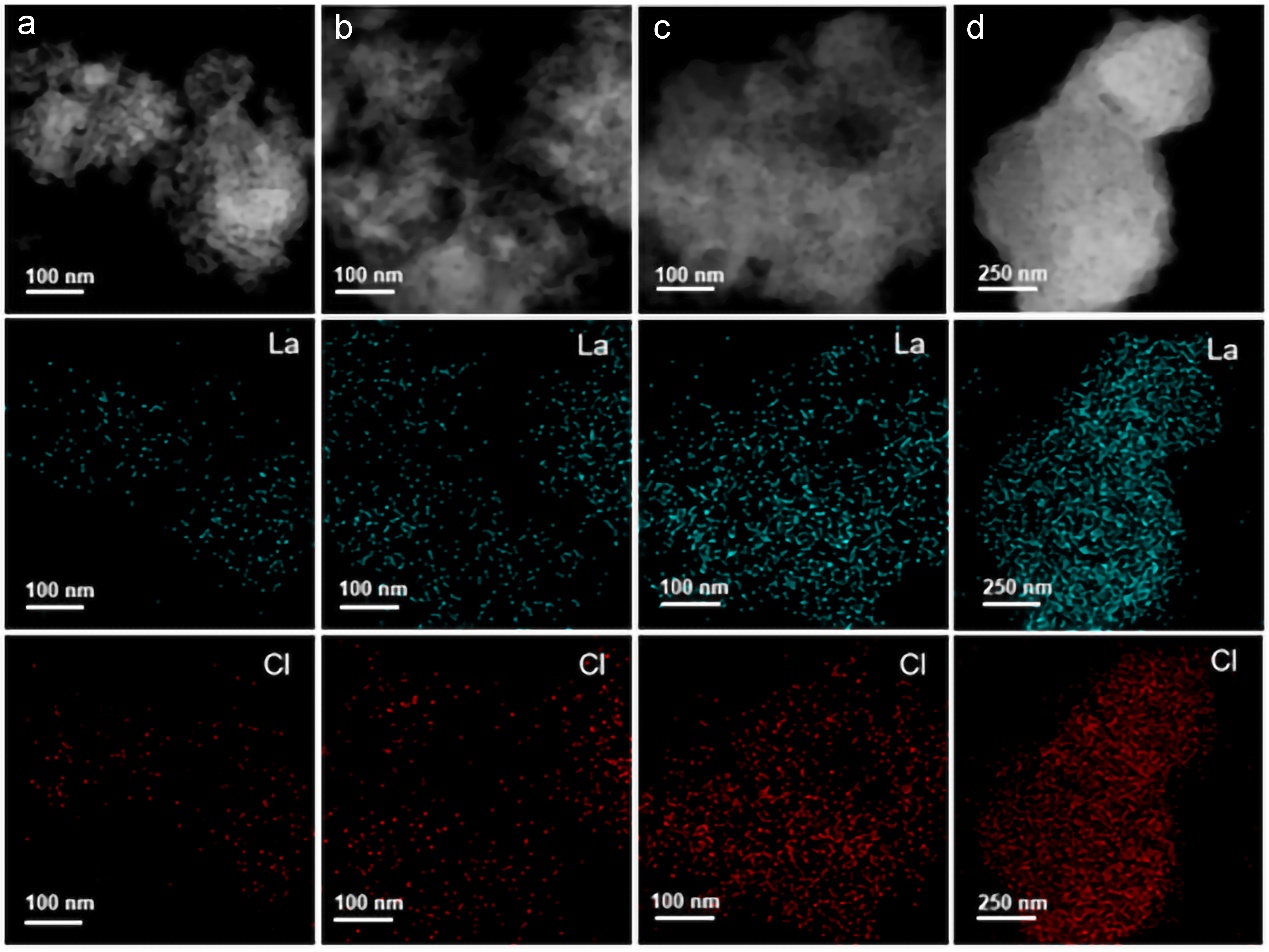


**Figure S5.** HRTEM images and corresponding elemental maps (La, Cl) of the 1.6% LaCl_3_/Al_2_O_3_ (**a**), 5.2% LaCl_3_/Al_2_O_3_ (**b**), 7.4% LaCl_3_/Al_2_O_3_ (**c**) and 9.2% LaCl_3_/Al_2_O_3_ (**d**) catalysts.


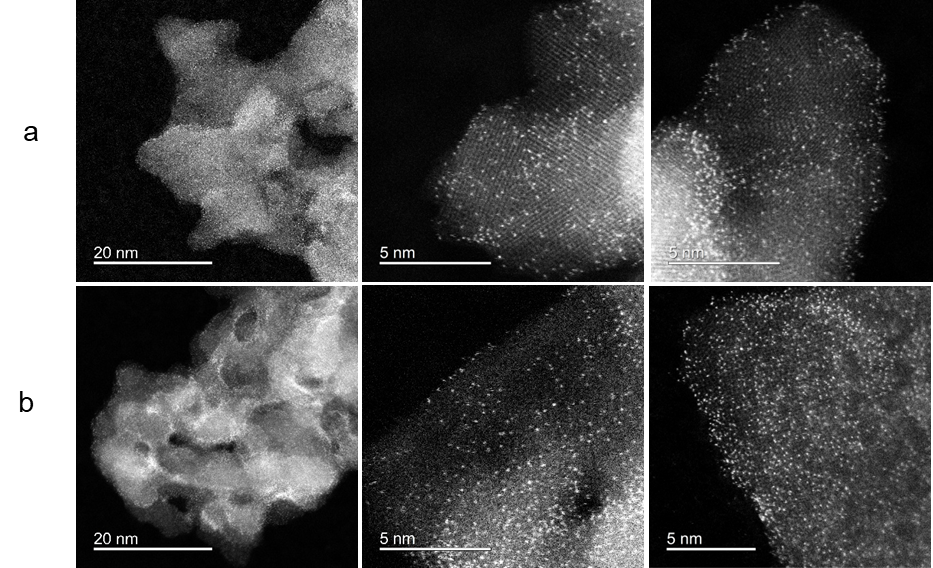


**Figure S6.** Atomic resolution STEM images of the 5.2% LaCl_3_/Al_2_O_3_ (**a**) and 7.4% LaCl_3_/Al_2_O_3_ (**b**) catalysts.


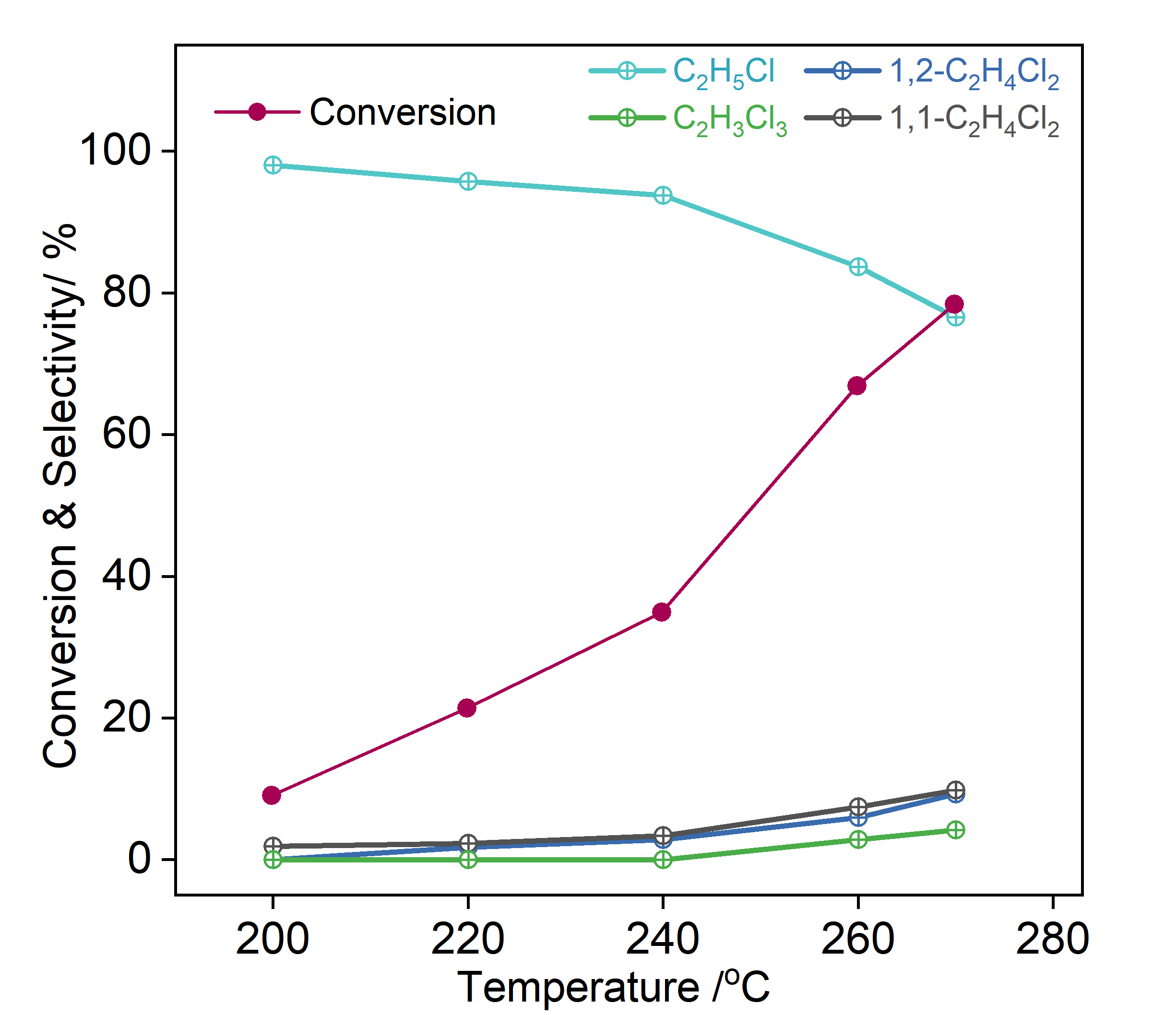


**Figure S7.** C_2_H_6_ conversion and selectivity as a function of temperature in the chlorination of C_2_H_6_ over the θ-Al_2_O_3_. Reaction conditions: catalyst = 0.5 g, C_2_H_6_:Cl_2_:N_2_= 4:9:87, WHSV = 2000 ml·h^-1^·g^-1^, 0.1 MPa, 200-270 °C.


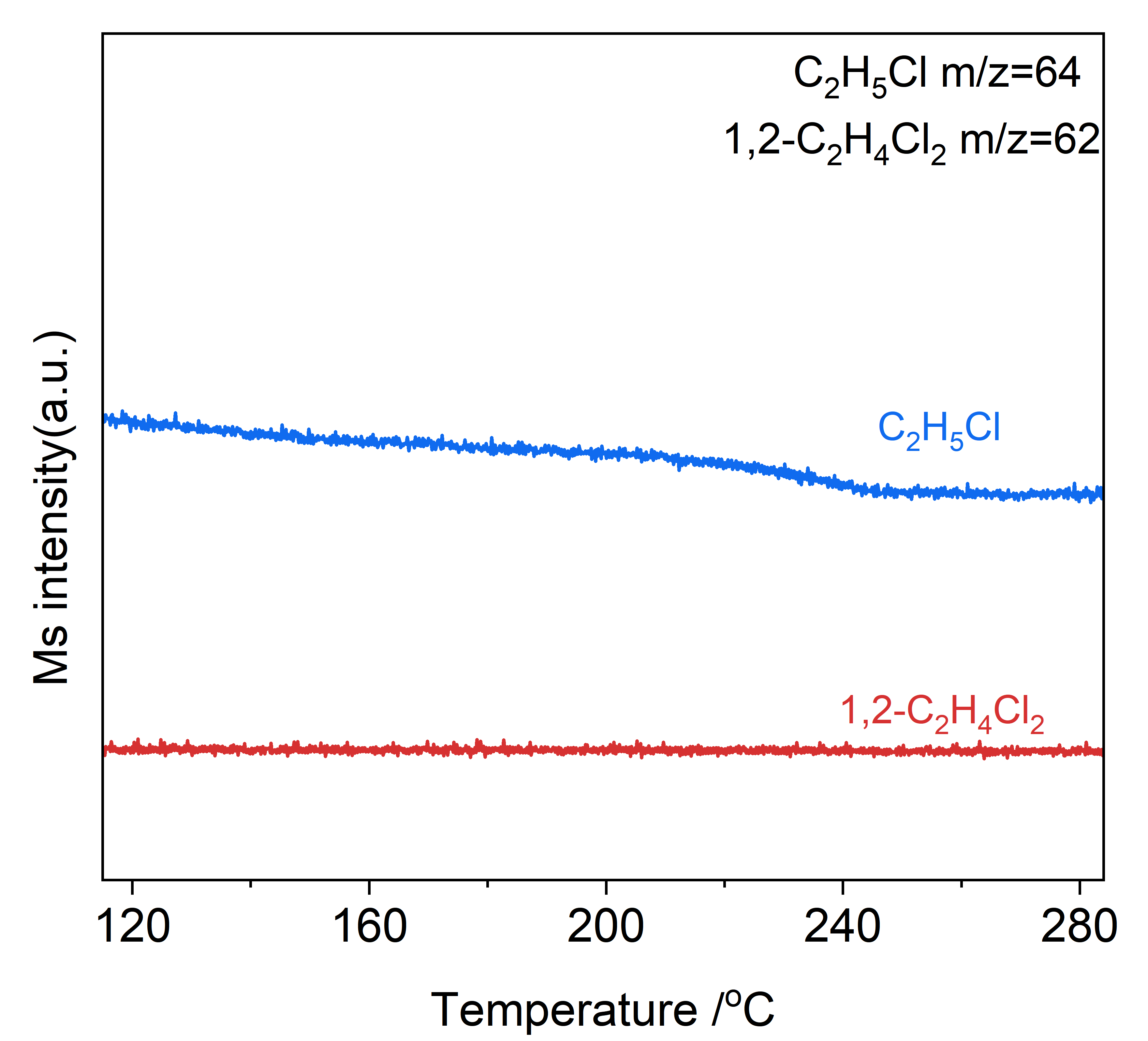


**Figure S8.** C_2_H_5_Cl-TPD over the 7.4% LaCl_3_/Al_2_O_3_ catalyst.


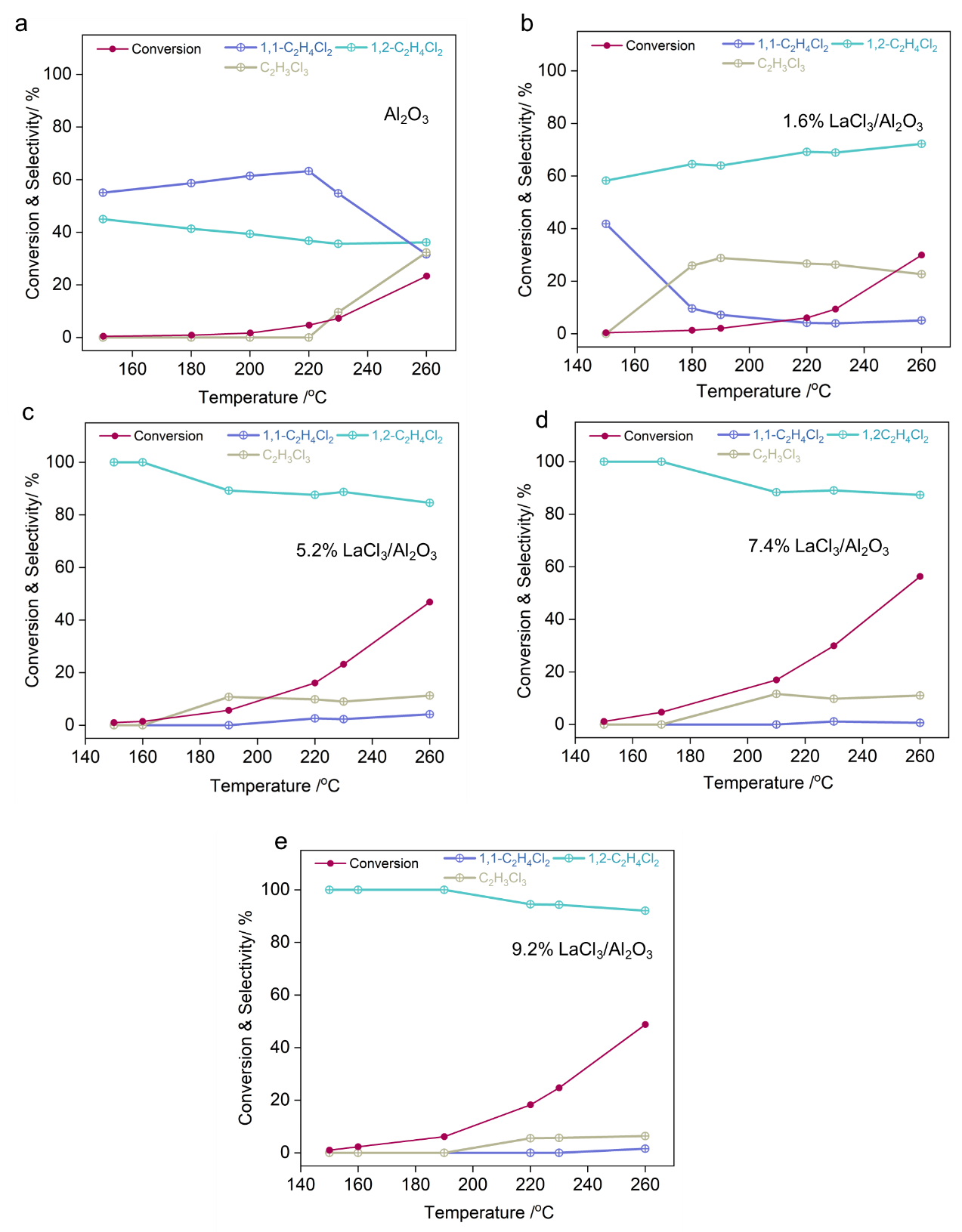


**Figure S9.** Conversion and selectivity as a function of temperature in the chlorination of C_2_H_5_Cl over the θ-Al_2_O_3_ (**a**), 1.6% LaCl_3_/Al_2_O_3_ (**b**), 5.2% LaCl_3_/Al_2_O_3_ (**c**), 7.4% LaCl_3_/Al_2_O_3_ (**d**) and 9.2% LaCl_3_/Al_2_O_3_ (**e**) catalyst. Reaction conditions: C_2_H_5_Cl/Cl_2_/N_2_ = 3.5:5:91.5, 150-260 °C, WHSV= 5500 ml·h^-1^·g^-1^.


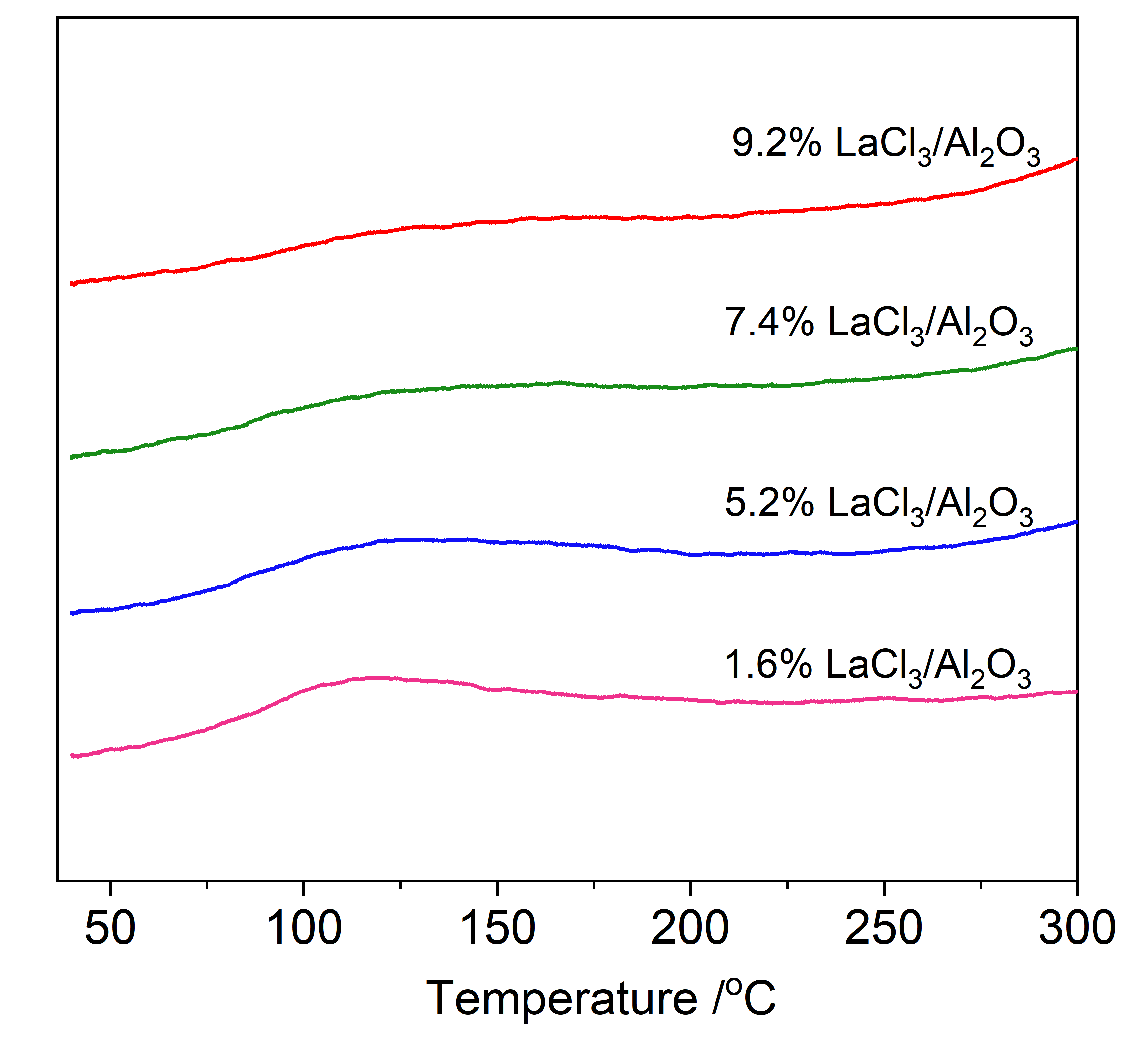


**Figure S10.** C_2_H_5_Cl-TPD of the LaCl_3_/Al_2_O_3_ catalysts with different La loadings.


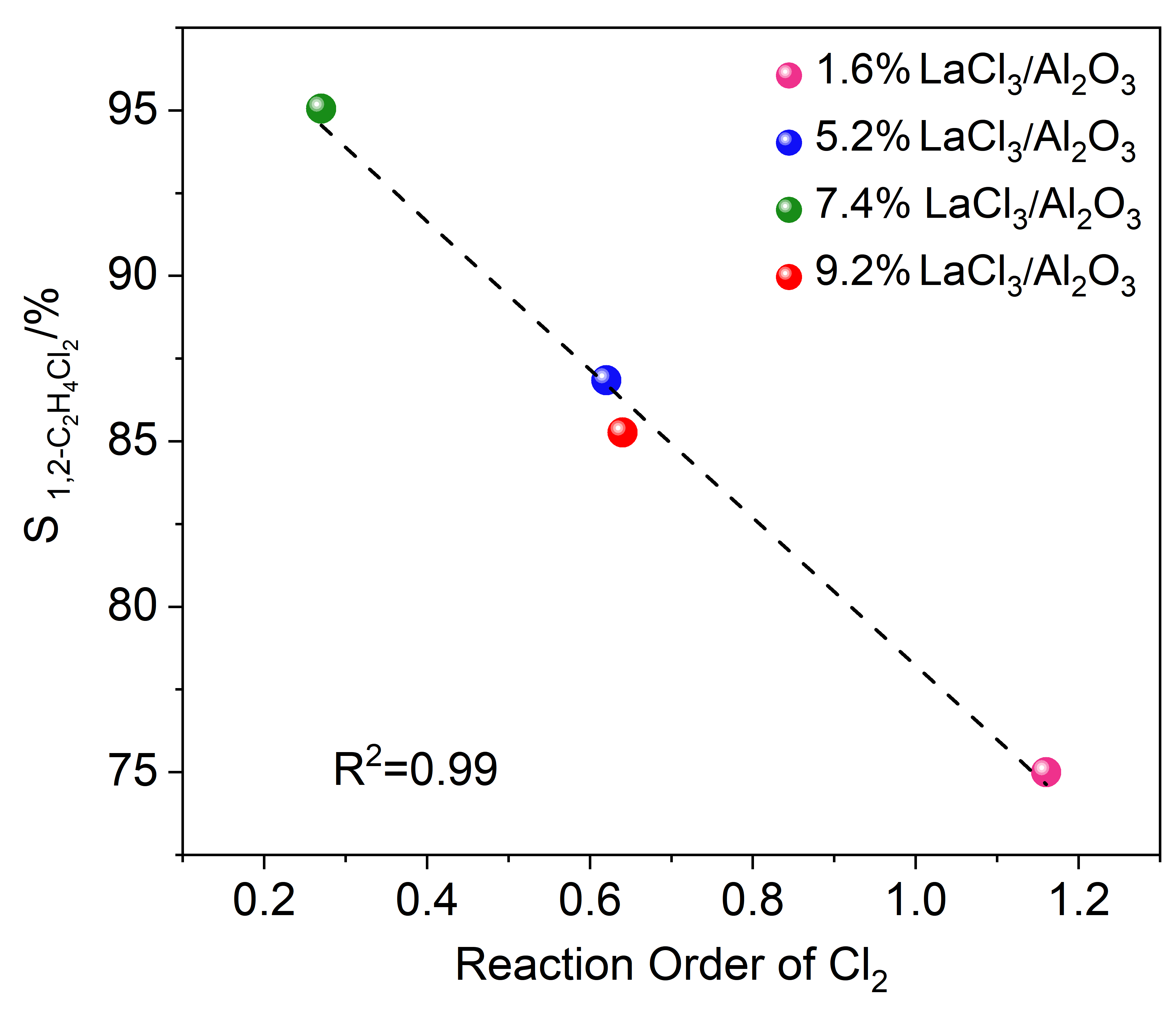


**Figure S11.** Selectivity to 1,2-C_2_H_4_Cl_2_ as a function of the reaction order of Cl_2_ in C_2_H_5_Cl chlorination over the LaCl_3_/Al_2_O_3_ catalysts. Reaction conditions: C_2_H_5_Cl:Cl_2_ = 1.5-4.5:2-5, 200-230 °C, WHSV = 6000-8000 ml·h^-1^·g^-1^.


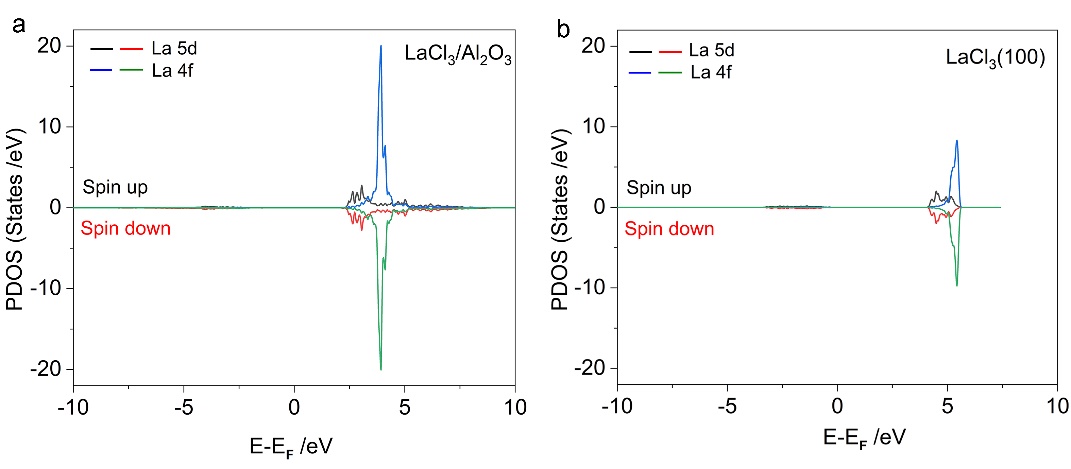


**Figure S12.** The PDOS for surface La sites in LaCl_3_/Al_2_O_3_ (**a**) and LaCl_3_(100) (**b**) models.


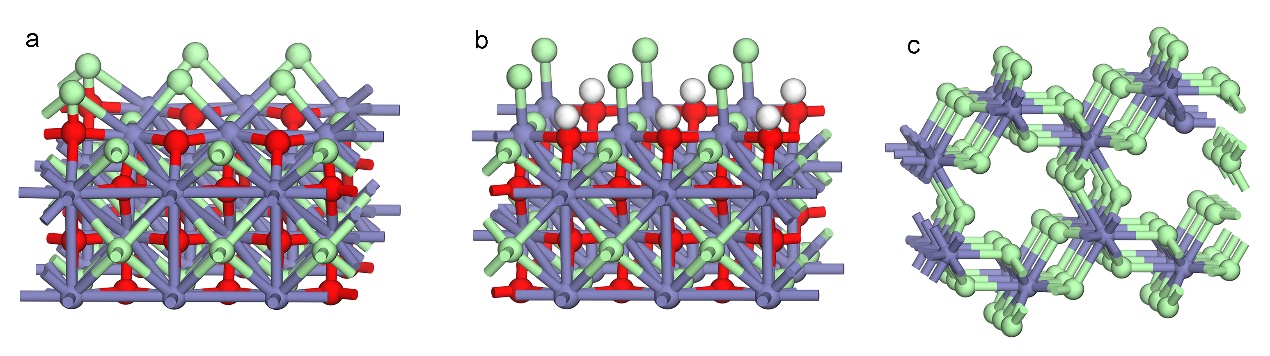
**Figure S13.** Cl-saturated LaOCl(100) (**a**), HCl-saturated LaOCl(100) (**b**) and LaCl_3_(100) (**c**) models.


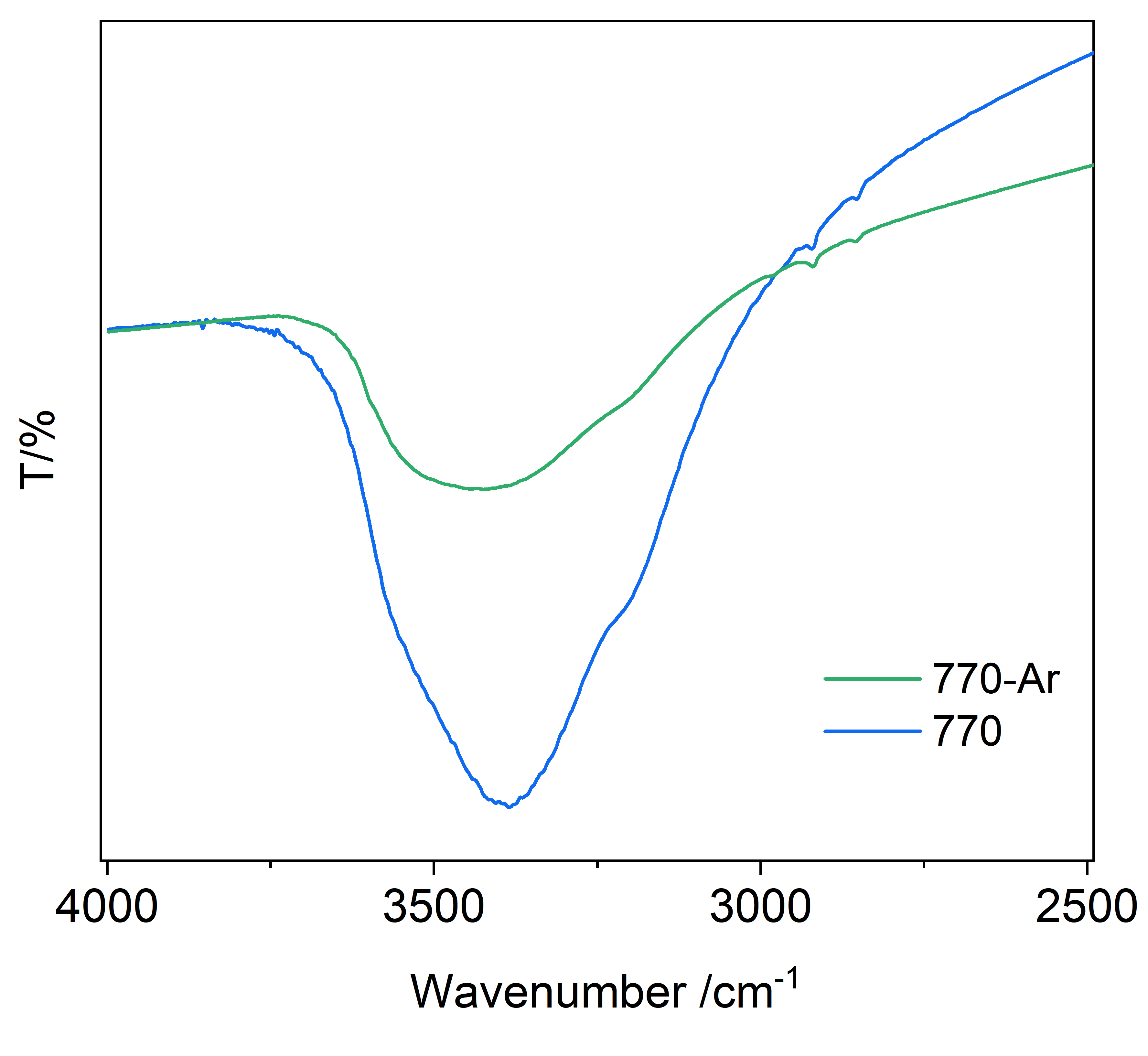


**Figure S14.** Transmission infrared spectra of La_2_O_3_ catalysts after 770 min of reaction and then being treated with Ar at 350 °C for 5 h.


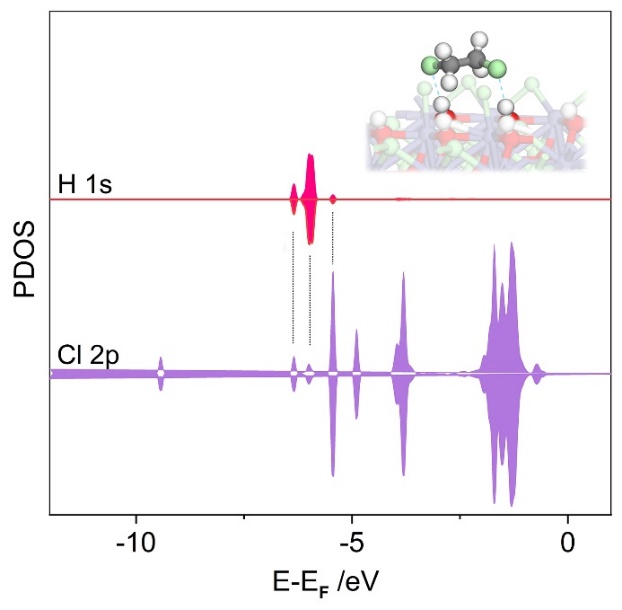


**Figure S15.** PDOS analysis of 1,2-C_2_H_4_Cl_2_ adsorbed on the hydroxyl-covered LaOCl model.

**Table S1.** The measured La content of LaCl_3_/Al_2_O_3_ catalysts quantified by ICP-OES.

| Sample | La /wt% |
| --- | --- |
| 1.6% LaCl_3_/Al_2_O_3_ | 1.62 |
| 5.2% LaCl_3_/Al_2_O_3_ | 5.24 |
| 7.4% LaCl_3_/Al_2_O_3_ | 7.38 |
| 9.2% LaCl_3_/Al_2_O_3_ | 9.20 |

**Table S2.** Orders of reaction determined from kinetic tests over the LaCl_3_/Al_2_O_3_ catalysts.

| Sample | C_2_H_5_Cl order | Cl_2_  order | Ea  (KJ·mol^-1^) |
| --- | --- | --- | --- |
| Al_2_O_3_ | 1.07±0.07 | 1.35±0.04 | 159.61±3.28 |
| 1.6% LaCl_3_/Al_2_O_3_ | 1.13±0.05 | 1.17±0.05 | 118.42±4.67 |
| 5.2%LaCl_3_/Al_2_O_3_ | 0.97±0.04 | 0.63±0.03 | 87.98±3.39 |
| 7.4% LaCl_3_/Al_2_O_3_ | 1.01±0.07 | 0.3±0.04 | 65.69±4.51 |
| 9.2% LaCl_3_/Al_2_O_3_ | 1.04±0.06 | 0.64±0.04 | 86.01±2.81 |

**Reference**

[1] Kresse, G. & Hafner, J. Ab initio molecular dynamics for liquid metals. *Phys. Rev. B* **47**, 558–561 (1993).

[2] Kresse, G. & Furthmuller, J. Efficiency of ab-initio total energy calculations for metals and semiconductors using a plane-wave basis set. *Comput. Mater. Sci*. **6**, 15–50 (1996).

[3] Kresse, G. & Furthmuller, J. Efficient iterative schemes for ab initio total-energy calculations using a plane-wave basis set. *Phys. Rev. B* **54**, 11169–11186 (1996).

[4] Perdew, J. P., Burke, K. & Ernzerhof, M. Generalized gradient approximation made simple. *Phys. Rev. Lett.* **77**, 3865–3868 (1996).

[5] Kresse, G. & Joubert, D. From ultrasoft pseudopotentials to the projector augmented-wave method. *Phys. Rev. B* **59**, 1758–1775 (1999).

[6] Henkelman, G., Uberuaga, B. P. & Jónsson, H. A climbing image nudged elastic band method for finding saddle pointsand minimum energy paths. *J. Chem. Phys.* **113,** 9901–9904 (2000).
